# Intrinsically stretchable three primary light-emitting films enabled by elastomer blend for polymer light-emitting diodes

**DOI:** 10.1126/sciadv.adh1504

**Published:** 2023-06-21

**Authors:** Min Woo Jeong, Jin Hyun Ma, Jae Seung Shin, Jun Su Kim, Guorong Ma, Tae Uk Nam, Xiaodan Gu, Seong Jun Kang, Jin Young Oh

**Affiliations:** ^1^ Department of Chemical Engineering (Integrated Engineering Program), Kyung Hee University, Yongin, Gyeonggi, 17104, Korea.; ^2^ Department of Advanced Materials Engineering for Information and Electronics, Kyung Hee University, Yongin, Gyeonggi, 17104, Korea.; ^3^ Integrated Education Institute for Frontier Science and Technology (BK21 Four), Kyung Hee University, Yongin, Gyeonggi, 17104, Korea.; ^4^ School of Polymer Science and Engineering, University of Southern Mississippi, Hattiesburg, MS 39406, USA.

## Abstract

Intrinsically stretchable light-emitting materials are crucial for skin-like wearable displays; however, their color range has been limited to green-like yellow lights owing to the restricted stretchable light-emitting materials (super yellow series materials). To develop skin-like full-color displays, three intrinsically stretchable primary light-emitting materials [red, green, and blue (RGB)] are essential. In this study, we report three highly stretchable primary light-emitting films made from a polymer blend of conventional RGB light-emitting polymers and a nonpolar elastomer. The blend films consist of multidimensional nanodomains of light-emitting polymers that are interconnected in an elastomer matrix for efficient light-emitting under strain. The RGB blend films exhibited over 1000 cd/m^2^ luminance with low turn-on voltage (<5 *V*_on_) and the selectively stretched blend films on rigid substrate maintained stable light-emitting performance up to 100% strain even after 1000 multiple stretching cycles.

## Teaser

Intrinsically stretchable full color light-emitting semiconductor films are achieved through elastomer blend for skin display.

## INTRODUCTION

Stretchable displays have garnered considerable attention as the next form of display to extend user-interactive applications (*1*–*3*). Polymer light-emitting diode (PLED) devices have led to the development of flexible displays. Recently, stretchable PLEDs have become a key component for high-resolution stretchable displays owing to their mechanical stretchability, high color purity, color tunability, resolution patterning, and power efficiency compared to previously reported stretchable light-emitting devices, such as light-emitting capacitors and electrochemical light-emitting devices (*4*, *5*).

To commercialize stretchable PLEDs, the development of stretchable light-emitting materials is essential for the production of a stretchable display, and various material concepts have been reported, which can be categorized into two: (i) extrinsically stretchable materials and (ii) intrinsically stretchable materials. Extrinsically stretchable PLEDs are based on structural strain engineering such as wrinkle/buckles, serpentine structures, and rigid islands (*6*–*18*). Although these approaches can be applied to conventional polymer light-emitting materials, there remain challenges in regard to light scattering under strain, device density, and strain accommodation on human skin.

In the case of intrinsic stretchability, substantial developments have been reported in molecular design, molecular interaction control, and nanoconfinement effects (*19*–*27*). Kim and Park (*22*) introduced intrinsically stretchable light-emitting polymer films based on molecular interaction engineering using plasticizers. They demonstrated that the intrinsic stretchability of a conventional light-emitting polymer (super yellow) can be enhanced by controlling the molecular coupling of the light-emitting polymer using a surfactant as a plasticizer. This leads to molecular sliding of entangled polymer chains, which improves the intrinsic stretchability of the conventional light-emitting polymer film with high luminance (>1000 cd/m^2^). However, the stretchable light-emitting materials developed by plasticizers are fundamentally not elastic but ductile, which can lead to irreversible molecular sliding and fatigue failure without an elastic substrate. Consequently, an elastic light-emitting film that is mechanically reliable under strain with high light-emitting performance is needed to further develop intrinsically stretchable PLEDs.

Polymer blends of light-emitting polymers and elastomers have been proposed as a promising strategy for providing elasticity to light-emitting polymer films (*28*–*30*). Nanoconfinement effect was reported to alter nonstretchable conjugated polymers to intrinsically stretchable nanoconfined elastomer spaces because the increased chain dynamics of the nanoconfined conjugated polymer in the elastomer matrix can significantly reduce its Young’s modulus (*20*). Zhang *et al.* (*28*) first applied the nanoconfinement effect to intrinsically stretchable light-emitting polymer films. The nanoconfined super yellow nanofibers in polyurethane exhibited high stretchability (100% strain) and higher luminance (15,631 cd/m^2^ at 0% strain on a rigid substrate) than previously reported intrinsically stretchable light-emitting films. Recently, Liu *et al.* (*29*) also reported intrinsically stretchable light-emitting films using a similar strategy based on a three-dimensional (3D) nanofiber penetrating network of super yellow in a polyacrylonitrile elastomer for better vertical charge transport. The elastic light-emitting film exhibited high stretchability (80% strain) and luminance (over 15,000 cd/m^2^ at 0% strain on a rigid substrate). Although these approaches have successfully achieved highly stretchable elastic light-emitting films, they have only obtained monochrome light-emitting devices using super yellow series materials because of the low miscibility of relatively nonpolar light-emitting polymers and polar elastomers, leading to severe phase separation (*28*, *29*).

To develop a fully colored display, three intrinsically stretchable primary light-emitting [red, green, and blue (RGB)] films are essential. Although previous studies have reported the expandability of blending with RGB light-emitting materials and an elastomer matrix, studies on stretchable RGB light-emitting films based on elastomer blends are still nascent (*28*). In addition, the advanced morphology of the blend film for improved light-emitting efficiency than the previously reported nanofiber-based blend film that prefers a horizontal current on the substrate is still being investigated. Thus, intrinsically stretchable and high-luminance RGB light-emitting films that prefer vertical currents are still necessary for stretchable full-color PLEDs.

Here, we present intrinsically stretchable RGB light-emitting films made from a blend of conventional RGB light-emitting polymers and nonpolar thermoplastic elastomers, which have an extremely reduced modulus and high stretchability compared to neat light-emitting polymers. The blend films were designed with low relative energy density (RED, < 1) and Flory-Huggins interaction parameter (χ) values for uniform blending while suppressing severe phase separation (*30*–*37*). The three primary light-emitting blend films have multidimensional nanodomains of light-emitting polymers in an elastomer matrix. The spherical nanodomains of light-emitting polymers are interconnected with 1D nanoweb and molecular network of light-emitting polymers, which efficiently recombine the injected electron and hole from electrodes even up to 100% strain without any mechanical damage, leading to strain-invariant high luminance of over 1000 cd/m^2^ (red, 1731 cd/m^2^; green, 1807 cd/m^2^; and blue, 2167 cd/m^2^ at 100% strain on a rigid substrate). This is a demonstration of intrinsically stretchable and mechanically durable RGB light-emitting films maintaining over 1000 cd/m^2^ at 100% strain up to 1000 multiple stretching cycles.

## RESULTS

**Fig. 1.**
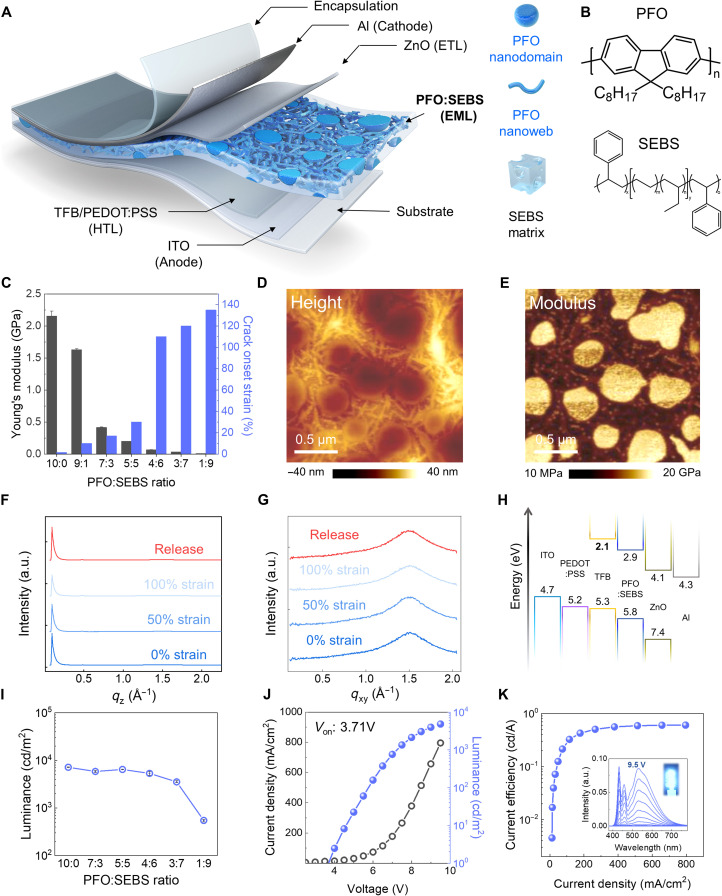
Intrinsically stretchable blend films of organic light-emitting polymer formed nanodomains and nanoweb network structure, improving stretchability and charge transport. (**A**) A schematic of structure and component layers of a polymer light-emitting diode (PLED) device. The PLED device consists of a poly(9,9-di-n-octylfluorenyl-2,7-diyl) (PFO):styrene-ethylene-butylene-styrene (SEBS) stretchable blend film, which has nanodomains and a nanoweb network structure. (**B**) Illustration of the chemical structures of PFO (blue light-emitting material) and SEBS (elastomer). (**C**) Young’s modulus and crack onset strain (COS) of PFO:SEBS blend films at different blending ratios measured using the buckling method and film on elastomer. (**D** and **E**) The atomic force microscopy (AFM) height and DMT modulus images of the PFO:SEBS blend film, respectively. The blend films were formed in the phase separation between nanodomains and nanoweb structures, as shown in the AFM images. (**F** and **G**) Illustration of the crystallinity extracted from grazing-incidence wide-angle x-ray scattering (GIWAX) analysis data of blend films under various strain conditions and the 1D diffraction intensity curves in two directions; out of plane and in-plane. (**H**) Energy band diagram of all the components in the proposed PLED device. Indium tin oxide (ITO) (anode), PEDOT:PSS (hole injection layer), TFB (hole transport layer), PFO:SEBS [emitting layer (EML)], ZnO (electron transport layer), and Al (cathode). (**I**) The luminance of PLED devices changes with varying PFO:SEBS blend ratios. The electroluminescence (EL) performance is almost maintained until 60% SEBS contents. (**J** and **K**) Illustration of the *J-V-L* characteristics of PLED with 4:6 PFO:SEBS emitting films with current efficiency-current density characteristics. The inset shows the EL spectra up to 9.5 V and a PLED device. a.u., arbitrary units.

Figure 1A shows the schematic of the PLED device structure. Light-emitting film was prepared by blending blue light-emitting polymer, poly(9,9-di-n-octylfluorenyl-2,7-diyl) (PFO) and styrene-ethylene-butylene-styrene (SEBS) elastomer (Fig. 1B). The PFO:SEBS blend ratio was optimized with changed crack onset strain and Young’s modulus of the films. Figure 1C shows the overall Young’s modulus and crack onset strain of the blend films as a function of the PFO:SEBS blend ratio. As increasing SEBS elastomer fraction, the Young’s modulus of the blend film significantly decreased to 415 MPa (7:3 blend ratio) from 1.63 GPa (9:1 blend ratio), and the value was saturated to 1:9 blend ratio (9 MPa). In addition, the crack onset strain of the blend film was significantly increased over 100% from 4:6 blend ratio compared to ~10% (9:1 blend ratio) (figs. S1 and S2), and the method of Young’s modulus extraction is shown in fig. S3. Considering the modulus and crack onset strain results, the 4:6 blend ratio was applied to stretchable blue light-emitting film.

Atomic force microscopy (AFM) was performed using the peak force quantitative nanomechanical mapping (QNM) method to investigate the morphology of the controlled blend film (PFO:SEBS, 4:6) at the nanoscale. Figure 1 (D and E) shows the height and modulus images of the blend film, respectively. The blend film exhibits multidimensional hybrid phase separation based on spherical and rod PFO nanodomains that are interconnected like nanoweb structures, which correspond to the modulus of neat PFO (~4 GPa; fig. S2). In principle, the phase separation of the polymer blend originates from the surface energy difference between PFO (36.3 mJ/m^2^) and SEBS (33.6 mJ/m^2^), which can be converted into the χ value (χ_PFO:SEBS_ 0.05 K) (fig. S4 and table S1) (*38*, *39*). In addition, the RED values of the PFO and SEBS blend systems are less than 1 (0.85), indicating that the materials are miscible with each other (fig. S5 and table S2). Blend films with various blend ratios (PFO:SEBS, 1:9 to 9:1) exhibited uniform nanophase separations, indicating that PFO and SEBS are partially miscible (fig. S6). The isolated PFO nanodomains were clearly interconnected at 40 weight % (wt %) PFO in the blend film, which facilitated both vertical and horizontal charge carrier transport under strain without mechanical damage. To better understand the nanostructure of the PFO and SEBS blend films, the crystallinity of the blend films was investigated using grazing-incidence wide-angle x-ray scattering (GIWAX). Figure 1 (F and G) exhibits the GIWAX spectra of the 4:6 blend films under 0, 50, 100% strain and released state. The blend film exhibited a similar crystallinity to that of the neat PFO film, indicating that the phase-separated PFO in the blend film has a typical crystal structure similar to that of the neat PFO film (figs. S7 and S8). In addition, the blend film preserved its initial crystallinity up to 100% strain without mechanical damages, demonstrating that the PFO crystal structure of the blend film was not affected by the applied strain. The dichroic ratio, which is the ratio of the polarized ultraviolet (UV)–visible absorption peak ratio between perpendicular and parallel lights indicating the molecular alignment of the conjugated polymer, is in agreement with these results (fig. S9). The dichroic ratio of the blend film maintained its initial value (1.02) up to 100% strain, indicating that the SEBS matrix is mainly stretched while preserving the PFO nanostructure under the applied strain.

On the basis of the morphology and crystallinity analysis results, we fabricated PLED devices with a PFO:SEBS blend film to evaluate their electroluminescence (EL) performance. The PLED device structure and its interfacial energy band alignment are designed for high luminance, as shown in Fig. 1H and fig. S10, and the detailed fabrication process is discussed in the experimental method. Figure 1J and fig. S11 show the characteristics of blue-emitting PLEDs in terms of current density, luminance, and applied voltage (*J*-*V*-*L*) as a function of the blend ratio. Light-emitting films with various blend ratios (PFO:SEBS) had maximum luminance values of 7081 (10:0), 6149 (7:3), 6471 (5:5), 5823 (4:6), 3777 (3:7), and 573 cd/m^2^ (1:9). In case of PLED using PFO:SEBS (4:6) blend film, the luminance value was maintained at approximately 82% compared to using neat PFO film, although the blend film is consisted of 60 wt % insulating SEBS. This result might be due to the percolation path of the PFO domain and the interconnected nanoweb, which can lead to dilution of the charge trap density in the blend film because the insulating matrix is known to reduce the charge trap density of conjugated polymers (*28*, *35*). To prove this assumption, time-resolved photoluminescence (PL), electron-only devices (EODs), and hole-only devices (HODs) were evaluated with neat and blend films (figs. S12 and S13). The average PL life time of neat and PFO:SEBS blend films exhibited a slight decrease from 0.33 to 0.30 ns. This result indicates that no additional traps were formed after the elastomer blend. The electron current density was slightly increased from 140 (neat PFO) to 407 mA/cm^2^ (PFO:SEBS blend film). For HODs, the hole current density was also elevated to 425 (4:6 blend ratio) from 130 mA/cm^2^ (neat PFO film). The diluted charge trap resulted in a stable current efficiency of 0.6 cd/A at 800 mA/cm^2^ (Fig. 1K), and the emission wavelength of the blend film was slightly dependent on the operating voltage (inset in Fig. 1K) (*24*).

**Fig. 2.**
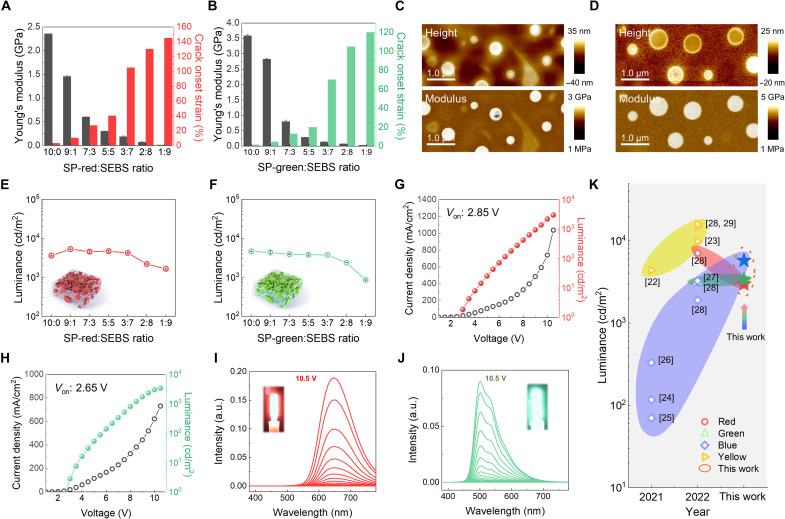
Expanding to red and green color stretchable polymer light-emitting diode (PLED) devices. (**A** and **B**) Illustration of the Young’s modulus and crack onset strain (COS) of spiro-red (SP-red) and spiro-green (SP-green) at varying styrene-ethylene-butylene-styrene (SEBS) blending ratios. (**C** and **D**) Atomic force microscopy (AFM) height (up) and DMT modulus [down, by peak force quantitative nanomechanical mapping (QNM) mode] images with 3:7 ratio for SP-red:SEBS films and 2:8 ratio for SP-green:SEBS films. (**E** and **F**) The luminance of red and green PLED devices with SP-red:SEBS and SP-green:SEBS blend films changed with the blend ratio. This is schematic illustration is inserted in each graph. (**G** and **H**) Current density and luminance versus voltage of PLEDs with 30% SP-red and 20% SP-green blend films. (**I** and **J**) Electroluminescence (EL) spectra of red and green PLED devices exhibited the same peak position during applied voltage sweep. (**K**) Advancement of various color light-emitting films in recent years.

To generalize this strategy to different primary color light-emitting conjugated polymers, two spiro-copolymers that emit red [spiro-red (SP-red)] and green [spiro-green (SP-green)] lights were applied to our blend system using a SEBS elastomer. The blend ratios of red and green light-emitting polymers and SEBS blend films were controlled by setting the Young’s modulus at a lower value than that of human skin and stretchability under 100% strain like the PFO:SEBS blending system (Fig. 2, A and B, and figs. S14 to S17). Both 3:7 (SP-red:SEBS) and 2:8 (SP-green:SEBS) blend films meet the requirements of the stretchability (over 100%) and Young’s modulus (below 10^2^ MPa, which is the average modulus range of human skin) for emitting red and green lights, respectively. In addition, shear stress and shear viscosity (η) of three primary color solutions as function of shear rate were measured for the kinetics of film formation (fig. S18). The viscosity values of the RGB blend solutions were comparable (green: 1.80, blue: 1.12, and red: 0.64 cP at 16/s, shear rate similarly to spin-coating speed), which influences the phase separation of the blend films. The QNM analysis provided the Young’s modulus of each component and material identification within the separated phases (Fig. 2, C and D). The SP-green:SEBS blend film exhibited nanodomain-based nanoweb phase separation, which is similar to the PFO:SEBS blend film, whereas the SP-red:SEBS blend film exhibited only nanodomain-based phase separation. These phase separations of the blend films are mainly caused by the surface energy difference between the light-emitting polymer and SEBS, which is illustrated in fig. S4 (*38*, *39*). The neat SP-green film exhibited a similar surface energy (38.6 mJ/m^2^) to PFO and SEBS, whereas the neat SP-red film exhibited higher surface energy (41.8 mJ/m^2^) than other films. With these surface energies, the χ values of the green and red blend films were determined as χ_SP-green:SEBS_ 0.17 K and χ_SP-red:SEBS_ 0.45 K, respectively (table S1). The higher χ_SP-red:SEBS_ value than others resulted in relatively further phase separation, which could be the reason for the only nanodomain-based phase separation of the red blend film. For a better understanding of phase separation, the nanoscale morphologies of red and green light-emitting films with different blend ratios (SP-red: SEBS and SP-green: SEBS; 9:1 to 1:9) were further analyzed using AFM. The red and green light-emitting films exhibited similar phase separation to blue light-emitting film as the SEBS content increased (figs. S19 and S20). To gain insight into the crystallinity of the green and red blend films, GIWAX analysis was performed, as shown in fig. S21. The GIWAX spectra demonstrated that all blend films maintained their initial crystallinity up to 100% strain, indicating that the elastic SEBS matrix mainly absorbs applied strain instead of the light-emitting polymer domains. The continuous dichroic ratios of the blend films during 100% stretching support the GIWAX results (figs. S22 and S23).

To verify this universal elastomer blending system for other light-emitting polymers, the EL performance of the red and green blend films was controlled by the blend ratio in the identical PLED structures (Fig. 2, E and F). The interfacial energy-band alignment details of the red and green PLEDs are shown in figs. S24 and S25. The main EL properties, such as luminance, current density, and current efficiency, were evaluated on an indium tin oxide (ITO)–glass rigid substrate. PLED devices with SP-red:SEBS blend film ratios in the range of 0 to 90 wt % SEBS exhibited maximum luminance values of 3698 (10:0), 5135 (9:1), 4907 (7:3), 4460 (5:5), 4061 (3:7), 2280 (2:8), and 1639 (1:9) cd/m^2^. For the same SEBS content variation of SP-green blend films, the devices exhibited maximum luminance values of 4746 (10:0), 4003 (9:1), 4060 (7:3), 4030 (5:5), 3756 (3:7), 2418 (2:8), and 886 (1:9) cd/m^2^ (figs. S26 and S27). The performance of the red and green PLED devices was almost the same as that of the neat film up to 70 wt % (red PLED) and 80 wt % (green PLED) SEBS. Last, we determined that the optimized blending ratios of SP-red and green blend films were 3:7 (SP-red:SEBS) and 2:8 (SP-green:SEBS), respectively, based on its high performance and sufficient stretchability. The *J*-*V*-*L* curves of the optimized red- and green-emitting PLED devices are shown in Fig. 2 (G and H), respectively. Both devices exhibit typical PLED *J*-*V*-*L* curves with low turn-on voltage (*V*_on_ at 1 cd/m^2^ luminance, red: 2.85 *V*_on_ and green: 2.65 *V*_on_). These results indicate that the red and green PLEDs have suitable band alignments for the efficient radiative recombination of the injected electrons and holes from the cathode and anode. Moreover, both SP-red:SEBS and SP-green:SEBS blend films exhibited slightly decreased PL lifetimes (fig. S28) and enhanced charge densities of electron and hole carriers compared to each neat film (fig. S29). The electron current densities of red and green blend films were boosted to 210 (SP-red:SEBS, 3:7) and 2180 (SP-green:SEBS, 2:8) mA/cm^2^ from 55 (neat SP-red) and 151 (neat SP-green) mA/cm^2^, respectively. For HODs, the hole current densities of red and green blend films were also increased from 29 (neat Red) and 6 (neat Green) mA/cm^2^ to 38 (red:SEBS, 3:7) and 17 (green:SEBS) mA/m^2^, respectively. The charge carrier transport of the light-emitting films was improved by blending with the SEBS elastomer due to the charge-trap dilution. However, the overall luminance values of all blend films were reduced as the SEBS content increased up to 90 wt %. These results indicate that the light-emitting performance is dominantly affected by absolute contents of light-emitting polymers in the blend films. The EL spectra of the red and green PLEDS are shown in Fig. 2 (I and J) as a function of the operating voltage. The peak positions of EL are 645 and 504 nm, without any peak shift. To compare our primary color luminance values of the blend films in the pristine state, the previously reported stretchable RGB color luminance values obtained using various material strategies are analyzed, as shown in Fig. 2K, and their Commission international de l’eclairage 1931 (CIE 1931) coordination (*x*,*y*) values are summarized in table S3 with the previously reported works.

**Fig. 3.**
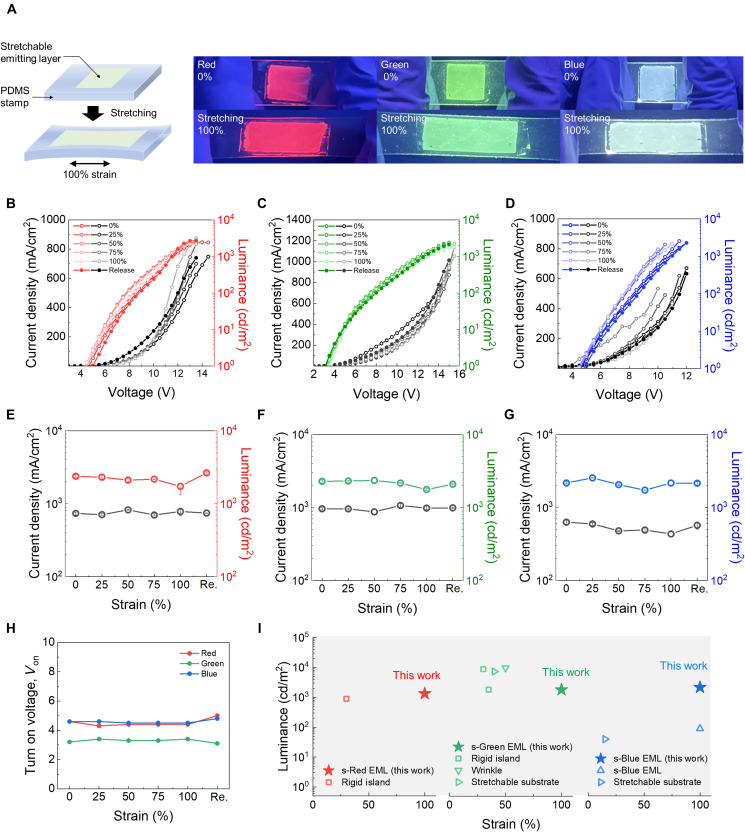
Stretchability of red, green, and blue (RGB) blend films and luminescence performance under various strain conditions. (**A**) Photoluminescence (PL) pictures of R, G, and B blend films on 0 and 100% strain excited under a 365-nm UV lamp. The measurement method was described in left side. (**B** to **D**) The *J-V-L* curves of polymer light-emitting diodes (PLEDs) with 3:7 spiro-red (SP-red):styrene-ethylene-butylene-styrene (SEBS), 2:8 spiro-green (SP-green):SEBS, and 4:6 poly(9,9-di-n-octylfluorenyl-2,7-diyl) (PFO):SEBS films under 0, 25, 50, 75, 100% and released strain. (**E** to **G**) The RGB PLED device’s current density and luminance values when the strain was applied. (**H**) The turn-on voltages (*V*_on_) under various strain conditions. (**I**) Comparison between previously reported results and the obtained stretchable three-primary color PLED devices performance under various strain conditions.

For systematic evaluation of the stretchability of the RGB blend films in PLEDs, the transfer-printing method was used to fabricate PLED devices because this study is focused on stretchable light-emitting materials. The selectively stretched blend film on polydimethylsiloxane (PDMS) stamps was transferred on rigid PLED substrates at various strain rates to isolate the strain effects of other layers. The transfer-printing method is shown in fig. S30. Figure 3A shows the real-time PL spectra of 100% stretched RGB blend films on PDMS stamps under 365-nm UV light. Each film clearly emitted RGB colors even under 100% strain without mechanical damages. Furthermore, we evaluated the EL performance of the stretched blend film at varying strain rates in the PLED device. Figure 3 (B to D) exhibits the *J*-*V*-*L* curves of the three primary color PLEDs with the stretched blend films on various strain conditions (0, 25, 50, 75, and 100%). The light-emitting films had similar thickness range (approximately 70 nm), and these luminance properties were less dependent on the film thickness (fig. S31) (*40*). Notably, each RGB PLED exhibited almost identical *J*-*V*-*L* curves up to 100% strain without significant electrical degradation, even after strain release. The RGB PLED has high initial luminance (>10^3^ cd/m^2^), and the values remained in the same order up to 100% strain at low turn on voltages (below 5 *V*_on_), as shown in Fig. 3 (E to H) and figs. S32 to S34. Our hypothesis is that the highly stable light-emitting performance under strain is due to nanodomain-based phase separation of the blend films. The isotropic light-emitting polymer nanodomains are not affected by the applied strain, as supported by the continuous dichroic ratio of the blend films regardless of strain. Moreover, the nanoweb or molecular level intermixed networks of light-emitting polymers in SEBS matrix can efficiently interconnect the light-emitting nanodomains on strain, which is desirable for vertical and parallel charge transport. In addition, the low *V*_on_ has advantage of low power consumption for wearable display applications (electronic skin and medical fields) with electrical long-term stability. The transfer-printed light-emitting films exhibit relatively lower luminance than spin-coated film on device substrate owing to inevitable mechanical damage during film transfer process (*20*). To the best of our knowledge, these values are the highest luminance values of stretchable RGB light-emitting film under 100% strain ever reported, as shown in Fig. 3I. With these EML films, we further fabricated flexible PLEDs and these EL performances were almost identical with the PLEDs fabricated on ITO/glass rigid substrates. In addition, the flexible PLEDs can be integrated with human finger and showed stable operation during bending-release motion (fig. S35 and movie S1).

**Fig. 4.**
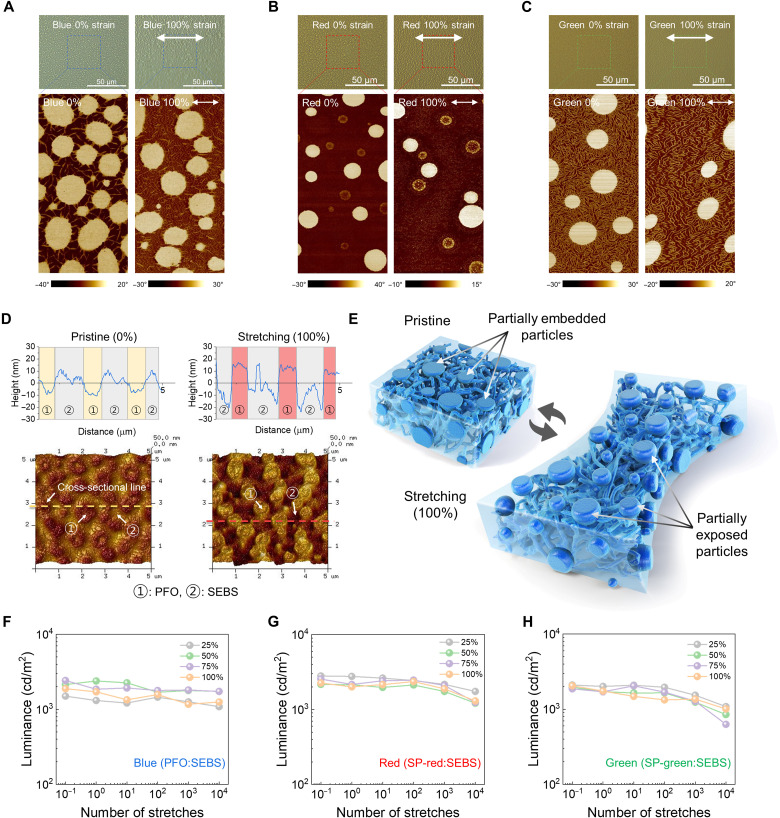
Mechanism proof and durability evaluation of red, green, and blue (RGB) blend films. (**A** to **C**) Optical microscope (OM) images (top) and atomic force microscopy (AFM) phase images (bottom) under 0 and 100% strain with blue, red, and green blend films. (**D**) A comparison of the morphological changes of the blend films in nanoscale before (left) and after stretching (right) using 1D cross-sectional height or 3D AFM analysis. (**E**) Schematic of the film morphology and in situ structural changes after stretching the films. (**F** to **H**) The luminance changes of polymer light-emitting diode (PLED) devices through multiple stretching cycles with poly(9,9-di-n-octylfluorenyl-2,7-diyl) (PFO):styrene-ethylene-butylene-styrene (SEBS), spiro-red (SP-red):SEBS, and spiro-green (SP-green):SEBS films. The blend films were stretched up to 25, 50, 75, and 100% strain on each cycle.

To uncover the reason for the highly stable light-emitting performance under strain, the micro- and nanoscale morphologies of the stretchable RGB films were analyzed under strain using an optical microscope (OM) and AFM analysis equipment (Fig. 4, A to C, and figs. S36 and S37). The average distances between nanodomains were lineally widened by the degree of stretching. The nanodomain-based phase separation of the all-blend films efficiently release the applied strain by stretching the elastic SEBS matrix instead of the light-emitting domains, which efficiently preserve the morphology of the light-emitting nanodomains interconnected with nanowebs in the SEBS matrix, resulting in highly strain-robust elastic light-emitting blend films. For direct observation of the energy dissipation mechanism under strain, 3D morphology of the PFO:SEBS blend film was measured through AFM. Figure 4D shows 3D height AFM images of the pristine, 0% strain (left), and 100% stretched (right) films. Numbers 1 and 2 indicate PFO and SEBS phases, respectively. The partially embedded PFO nanodomains were gradually exposed up to 100% strain without morphological damages, which is in agreement with the proposed energy dissipation mechanism. This mechanism is generally applied to SP-red and SP-green blend films (fig. S38). Figure 4E illustrates the optical, electrical, and morphological results of the energy dissipation mechanism. The RGB light-emitting blend films are highly durable on multiple stretching cycles. Figure 4 (F to H) shows the luminance of the RGB blend films after multiple stretching up to 10,000 times on a rigid substrate. All RGB PLED devices exhibited over 1000 cd/m^2^ luminance for 1000 multiple stretching cycles at 25, 50, 75, and 100% strain and even operated up to 10,000 times at all strain ranges without mechanical damages (figs. S39 to S51), demonstrating that these blend films have the highest mechanical durability compared to previously reported results, as shown in fig. S52. Last, these elastic RGB blend films can be biaxially stretched up to 30% while maintaining a luminance of over 1000 cd/m^2^, which is a demonstration of the biaxially stretchable elastic light-emitting films for skin-like PLEDs (fig. S53).

## DISCUSSION

We fabricated three highly stretchable and robust primary color light-emitting films by blending light-emitting conjugated polymers and nonpolar elastomers for intrinsically stretchable PLED devices. This blend system has a multidimensional phase separation with nanodomain and nanoweb hybrid networks, which enables isotropic charge transport regardless of strain, resulting in efficient charge transport and recombination for high-luminance stretchable PLED without optoelectrical degradation. The three primary colors (red, green, and blue) blend films exhibited a high luminance value (>1000 cd/m^2^) up to 100% strain at low *V*_on_ (<5 V) and these values have even maintained for 10,000 multiple stretching cycles. We believe that this contribution will inspire researchers to pave the way for highly efficient skin-like stretchable displays.

## MATERIALS AND METHODS

### Materials

PFO (product no. 571652, blue), red-light-emitting spiro-copolymer (SPR-001, product no. 900444, average molecular weight 180,000, red), green light-emitting spiro-copolymer (SPG-01T, product no. 900441, green), p-Xylene, anhydrous toluene, anhydrous chloroform, 2-propanol (IPA), trichloro(octadecyl)silane (OTS), and Triton X-100 were purchased from Sigma-Aldrich. PEDOT:PSS (CLEVIOSTM PVP AI4083) was purchased from Heraeus Epurio. SEBS (H1062) was provided by AsahiKASEI. ZnO was obtained from Lumtec. Poly[(9,9-dioctylfluorenyl-2,7-diyl)-*co*-(4,4-(4-sec-butylphenyl)diphenylamine)] (TFB) was purchased from Avantama. PDMS (Sylgard, 184) and cross-linker agents were obtained from Hana Technology. Silver (Ag, 99.99%, 3- to 5-mm granules) and aluminum (Al, 99.999%, 3 mm by 3 mm pellets) were purchased from Syscience. Silicon wafers (formed 300-nm silicon oxide layer) were purchased from iTASCO. Patterned ITO substrates with a sheet resistance of 15 ohmsq^−1^ and bare glass substrates (20 mm by 20 mm) were purchased from AMG Korea. All the chemicals were used as received without further purification.

### Fabrication of red, green, and blue PLED on ITO substrates

The patterned ITO substrate was sequentially cleaned with deionized (DI) water, acetone, and isopropyl alcohol for 15 min using an ultra-sonicator (DH.WUC.A03H, DAIHAN Scientific). Thereafter, the substrate was treated with UV-ozone (UVC-150, Omniscience) for 15 min to increase hydrophilicity. PEDOT:PSS (hole injection layer) dispersed in DI water was spin-coated onto the substrate at 3000 rpm for 30 s and annealed at 150°C for 15 min in a vacuum oven. Next, a 1 wt % TFB solution for the hole transport layer was prepared by dissolving the TFB precursor in p-Xylene, and the solution was subsequently stirred at 80°C for 1 hour. This solution was spin-coated onto the PEODT:PSS film at 3000 rpm for 30 s and annealed at 180°C for 30 min in a vacuum oven. Three colors of stretchable R, G, and B light-emitting polymer solutions were prepared by blending light-emitting polymers and SEBS in toluene (red:3:7, green:2:8, and blue:4:6 blending ratio), and these solutions were stirred at 80°C for 2 hours. Each solution was fabricated at a concentration of 1 wt %. These solutions were spin-coated onto the TFB film at 1000 rpm for 60 s and annealed at 130°C for 30 min in a vacuum oven. Furthermore, a stretchable emitting layer (EML) was formed using the transfer printing method from an OTS-treated silicon wafer to an ITO substrate (this will be explained in detail in the next section). In addition, a ZnO electron transport layer (ETL) was spin-coated at 2000 rpm for 60 s and thereafter annealed at 60°C for 10 min in a vacuum oven. The entire procedure was conducted under vacuum or inert conditions. Last, 130 nm of aluminum electrode was deposited using a thermal evaporator.

### Transfer of stretched EML films to PLED devices

OTS SAM-treated silicon wafers were prepared for easy detachment of the films. First, the OTS solution was prepared by dissolving OTS liquid in anhydrous chloroform (5 mg/ml). This solution was spin-coated at 2000 rpm for 60 s on a silicon wafer, which was treated with O_2_ plasma in the plasma etching (PE) mode for 60 s. The spin-coated silicon wafer was annealed in ammonium hydroxide vapor (25°C) for 4 hours. The as-prepared EML solutions were spin-coated on an OTS silicon wafer at 1000 rpm for 60 s and annealed at 130°C for 30 min in a vacuum oven. When the films were completely prepared, they were transferred to PDMS (PDMS:cross-linking agent, 10:1 weight ratio, cured at 60°C overnight) using a quick detaching process. The transferred films on PDMS were stretched under various strain conditions, such as 0, 25, 50, 75, and 100%, and transferred again to PLED devices.

### Fabrication of EODs and HODs

The EODs and HODs had the following device structure, respectively; ITO (glass)/light-emitting polymer:SEBS/ZnO/Al (EOD) and ITO (glass)/PEDOT:PSS/TFB/light-emitting polymer:SEBS/PEDOT:PSS/Ag (HOD). All device components were spin-coated and annealed under the optimized conditions mentioned above. In addition, 0.25 wt % Triton X-100 was added to PEDOT:PSS as the electron barrier layer on the HOD to obtain a uniform film. In comparison with the Al electrode, the Ag electrode on the HOD was deposited up to 70 nm at a deposition rate of with 2.0 Å/s.

### Characterization

The EL and current-voltage-luminance characteristics of the PLEDs were measured using an *J*-*V*-*L* measurement system (M6100, McScience) connected to a source-meter unit (K-2400, Keithley) and spectroradiometer (CS-2000, Konica Minolta). PL spectra of the stretchable EML were captured under a 365-nm UV lamp (VL-6. LC, Vilber Lourmat). GIWAXS of polymeric thin films on silicon wafer were performed on Xeuss 2.0 (Xenocs Inc) with an x-ray wavelength of 1.54 Å, sample to detector distance of 15 cm, and incidence angle of 0.2°. Samples were kept under vacuum to minimize air scattering. Diffraction images were recorded on a Pilatus 1M detector (Dectris Inc.) and processed using the Nika software package, in combination with WAXSTools wavemetrics Igor.

## References

[1] J. Y. Oh, Z. Bao, Second skin enabled by advanced electronics. Adv. Sci. 6, 1900186 (2019). 10.1002/advs.201900186 PMC6548954 31179225

[2] S. Wang, J. Y. Oh, J. Xu, H. Tran, Z. Bao, Skin-inspired electronics: An emerging paradigm. Acc. Chem. Res. 51, 1033–1045 (2018). 29693379 10.1021/acs.accounts.8b00015

[3] Z. Zhang, Z. Bao, High luminescent polymers for stretchable displays. Natl. Sci. Rev. 10, nwac093 (2022). 36684509 10.1093/nsr/nwac093 PMC9843124

[4] D. Hu, X. Xu, J. Miao, O. Gidron, H. Meng, A stretchable alternating current electroluminescent fiber. Materials 11, 184 (2018). 29364836 10.3390/ma11020184 PMC5848881

[5] Z. Zhang, X. Shi, H. Lou, X. Cheng, Y. Xu, J. Zhang, Y. Li, L. Wang, H. Peng, A one-dimensional soft and color-programmable light-emitting device. J. Mater. Chem. C 6, 1328–1333 (2018).

[6] J. Liang, L. Li, X. Niu, Z. Yu, Q. Pei, Elastomeric polymer light-emitting devices and displays. Nat. Photonics 7, 817–824 (2013).

[7] D. K. Choi, D. H. Kim, C. M. Lee, H. Hafeez, S. Sarker, J. S. Yang, H. J. Chae, G.-W. Jeong, D. H. Choi, T. W. Kim, S. Yoo, J. Song, B. S. Ma, T.-S. Kim, C. H. Kim, H. J. Lee, J. W. Lee, D. Kim, T.-S. Bae, S. M. Yu, Y.-C. Kang, J. Park, K.-H. Kim, M. Sujak, M. Song, C.-S. Kim, S. Y. Ryu, Highly efficient, heat dissipating, stretchable organic light-emitting diodes based on a MoO_3_/Au/MoO_3_ electrode with encapsulation. Nat. Commun. 12, 2864 (2021). 34001906 10.1038/s41467-021-23203-y PMC8128878

[8] J. Liang, L. Li, K. Tong, Z. Ren, W. Hu, X. Niu, Y. Chen, Q. Pei, Silver nanowire percolation network soldered with graphene oxide at room temperature and its application for fully stretchable polymer light-emitting diodes. ACS Nano 8, 1590–1600 (2014). 24471886 10.1021/nn405887k

[9] T. Sekitani, H. Nakajima, H. Maeda, T. Fukushima, T. Aida, K. Hata, T. Someya, Stretchable active-matrix organic light-emitting diode display using printable elastic conductors. Nat. Mater. 8, 494–499 (2009). 19430465 10.1038/nmat2459

[10] Z. Yu, X. Niu, Z. Liu, Q. Pei, Intrinsically stretchable polymer light-emitting devices using carbon nanotube-polymer composite electrodes. Adv. Mater. 23, 3989–3994 (2011). 21796688 10.1002/adma.201101986

[11] S. Ganesh, R. Bade, X. Shan, P. T. Hoang, J. Li, T. Geske, L. Cai, Q. Pei, C. Wang, Z. Yu, Stretchable light-emitting diodes with organometal-halide-perovskite–polymer composite emitters. Adv. Mater. 29, 1607053 (2017). 10.1002/adma.201607053 28387463

[12] M. S. Lim, M. Nam, S. Choi, Y. Jeon, Y. H. Son, S.-M. Lee, K. C. Choi, Two-dimensionally stretchable organic light-emitting diode with elastic pillar arrays for stress relief. Nano Lett. 20, 1526–1535 (2020). 31990561 10.1021/acs.nanolett.9b03657

[13] M. S. White, M. Kaltenbrunner, E. D. Glowacki, K. Gutnichenko, G. Kettlgruber, I. Graz, S. Aazou, C. Ulbricht, D. A. M. Egbe, M. C. Miron, Z. Major, M. C. Scharber, T. Sekitani, T. Someya, S. Bauer, N. S. Sariciftci, Ultrathin, highly flexible and stretchable PLEDs. Nat. Photonics 7, 811–816 (2013).

[14] D. Yin, J. Feng, N. Jiang, R. Ma, Y. Liu, H.-B. Sun, Two-dimensional stretchable organic light-emitting devices with high efficiency. ACS Appl. Mater. Interfaces 8, 31166–31171 (2016). 27790909 10.1021/acsami.6b10328

[15] S. Jeong, H. Yoon, B. Lee, S. Lee, Y. Hong, Distortion-free stretchable light-emitting diodes via imperceptible microwrinkles. Adv. Mater. Technol. 5, 2000231 (2020).

[16] S. Choi, W. Jo, Y. Jeon, S. Kon, J. H. Kwon, Y. H. Son, J. Kim, J. H. Park, H. Kim, H. S. Lee, M. Nam, E. G. Jeong, J. B. Shin, T.-S. Kim, K. C. Choi, Multi-directionally wrinkle-able textile OLEDs for clothing-type displays. npj Flex. Electron. 4, 33 (2020).

[17] H. Hafeez, Z. Zou, D. H. Kim, J. Y. Shin, M. Song, C.-S. Kim, W. J. Choi, J. Song, J. Xiao, S. Y. Ryu, Multiaxial wavy top-emission organic light-emitting diodes on thermally prestrained elastomeric substrates. Org. Electron. 48, 314–322 (2017).

[18] Y. Lee, J. W. Chung, G. H. Lee, H. Kang, J.-Y. Kim, C. Bae, H. Yoo, S. Jeong, H. Cho, S.-G. Kang, J. Y. Jung, D.-W. Lee, S. Gam, S. G. Hahm, Y. Kuzumoto, S. J. Kim, Z. Bao, Y. Hong, Y. Yun, S. Kim, Standalone real-time health monitoring patch based on a stretchable organic optoelectronic system. Sci. Adv. 7, eabg9180 (2021). 34088675 10.1126/sciadv.abg9180 PMC8177712

[19] J. Y. Oh, S. Rondeau-Gagné, Y.-C. Chiu, A. Chortos, F. Lissel, G.-J. N. Wang, B. C. Schroeder, T. Kurosawa, J. Lopez, T. Katsumata, J. Xu, C. Zhu, X. Gu, W.-G. Bae, Y. Kim, L. Jin, J. W. Chung, J. B.-H. Tok, Z. Bao, Intrinsically stretchable and healable semiconducting polymer for organic transistors. Nature 539, 411–415 (2016). 27853213 10.1038/nature20102

[20] J. Xu, S. Wang, G.-J. N. Wang, C. Zhu, S. Luo, L. Jin, X. Gu, S. Chen, V. R. Feig; J. W. F. To, S. Rondeau-Gagné, J. Park, B. C. Schroeder, C. Lu, J. Y. Oh, Y. Wang, Y.-H. Kim, H. Yan, R. Sinclair, D. Zhou, G. Xue, B. Murmann, C. Linder, W. Cai, J. B.-H. Tok, J. W. Chung, Z. Bao, Highly stretchable polymer semiconductor films through the nanoconfinement effect. Science 355, 59–64 (2017). 28059762 10.1126/science.aah4496

[21] J. Y. Oh, D. Son, T. Katsumata, Y. Lee, Y. Kim, J. Lopez, H.-C. Wu, J. Kang, J. Park, X. Gu, J. Mun, N. G.-J. Wang, Y. Yin, W. Cai, Y. Yun, J. B.-H. Tok, Z. Bao, Stretchable self-healable semiconducting polymer film for active-matrix strain-sensing array. Sci. Adv. 5, eaav3097, (2019). 10.1126/sciadv.aav3097 PMC6839939 31723597

[22] J.-H. Kim, J.-W. Park, Intrinsically stretchable organic light-emitting diodes. Sci. Adv. 7, eabd9715 (2021). 33627424 10.1126/sciadv.abd9715 PMC7904263

[23] H. Zhou, S. J. Han, A. K. Harit, D. H. Kim, D. Y. Kim, Y. S. Choi, H. Kwon, K.-N. Kim, G.-T. Go, H. J. Yun, B. H. Hong, M. C. Suh, S. Y. Ryu, H. Y. Woo, T.-W. Lee, Graphene-based intrinsically stretchable 2D-contact electrodes for highly efficient organic light-emitting diodes. Adv. Mater. 34, 2203040 (2022). 10.1002/adma.202203040 35697021

[24] M. Ni, X. An, L. Bai, K. Wang, J. Cai, S. Wang, L. He, M. Xu, H. Liu, J. Lin, X. Ding, C. Yin, W. Huang, Intrinsically stretchable and stable ultra-deep-blue fluorene-based polymer with a high emission efficiency of ≈ 90% for polymer light-emitting devices with a CIE*_y_* = 0.06. Adv. Funct. Mater. 32, 2106564 (2022).

[25] C.-C. Jao, J.-R. Chang, C.-Y. Ya, W.-C. Chen, C.-J. Cho, J.-H. Lin, Y.-C. Chiu, Y. Zhou, C.-C. Kuo, Novel stretchable light-emitting diodes based on conjugated-rod block elastic-coil copolymers. Polym. Int. 70, 426–431 (2021).

[26] D.-H. Jiang, B. J. Ree, T. Isono, X.-C. Xia, L.-C. Hsu, S. Kobayashi, K. H. Ngoi, W.-C. Chen, C.-C. Jao, L. Veeramuthu, T. Satoh, S. H. Tung, C.-C. Kuo, Facile one-pot synthesis of rod-coil bio-block copolymers and uncovering their role in forming the efficient stretchable touch-responsive light emitting diodes. Chem. Eng. J. 418, 129421 (2021).

[27] X.-C. Li, L. Yao, W. Song, F. Liu, Q. Wang, J. Chen, Q. Xue, W.-Y. Lai, Intrinsically stretchable electroluminescent elastomers with self-confinement effect for highly efficient non-blended stretchable OLEDs. Angew. Chem. Int. Ed. 62, e202213749 (2023). 10.1002/anie.202213749 36350657

[28] Z. Zhang, W. Wang, Y. Jiang, Y.-X. Wang, Y. Wu, J.-C. Lai, S. Niu, C. Xu, C.-C. Shih, C. Wang, H. Yan, L. Galuska, N. Prine, H.-C. Wu, D. Zhong, G. Chen, N. Matsuhisa, Y. Zheng, Z. Yu, Y. Wang, R. Dauskardt, X. Gu, J. B.-H. Tok, Z. Bao, High-brightness all-polymer stretchable LED with charge-trapping dilution. Nature 603, 624–630 (2022). 35322250 10.1038/s41586-022-04400-1

[29] Y. Liu, M. Zhu, J. Sun, W. Shi, Z. Zhao, X. Wei, X. Huang, Y. Guo, Y. Liu, A self-assembled 3D penetrating nanonetwork for high-performance intrinsically stretchable polymer light-emitting diodes. Adv. Mater. 34, 2201844 (2022). 10.1002/adma.202201844 35488389

[30] K.-H. Jeon, J.-W. Park, Light-emitting polymer blended with elastomers for stretchable polymer light-emitting diodes. Macromolecules 55, 8311–8320 (2022).

[31] D. Liu, Z. Ding, Y. Wu, S. F. Liu, Y. Han, K. Zhao, In situ study of molecular aggregation in conjugated polymer/elastomer blends toward stretchable electronics. Macromolecules 55, 297–308 (2022).

[32] D. T. Duong, B. Walker, J. Lin, C. Kim, J. Love, B. Purushothaman, J. E. Anthony, T.-Q. Nguyen, Molecular solubility and Hansen solubility parameters for the analysis of phase separation in bulk heterojunctions. J Polym Sci B 50, 1405–1413 (2012).

[33] F. Machui, S. Abbott, D. Waller, M. Koppe, C. J. Brabec, Determination of solubility parameters for organic semiconductor formulations. Macromol. Chem. Phys 212, 2159–2165 (2011).

[34] C. M. Hansen, *Hansen Solubility Parameters* (CRC Press, 2007).

[35] A. Kunz, P. W. M. Blom, J. J. Michels, Charge carrier trapping controlled by polymer blend phase dynamics. J. Mater. Chem. C 5, 3042–3048 (2017).

[36] Z. Cao, J. Chen, S. Liu, M. Qin, T. Jia, J. Zhao, Q. Li, L. Ying, Y.-P. Cai, X. Lu, F. Huang, Y. Cao, Understanding of imine substitution in wide-bandgap polymer donor-induced efficiency enhancement in all-polymer solar cells. Chem. Mater. 31, 8533–8542 (2019).

[37] X. Xu, L. Yu, H. Yan, R. Li, Q. Peng, Highly efficient non-fullerene organic solar cells enabled by a delayed processing method using a non-halogenated solvent. Energ. Environ. Sci. 13, 4381–4388 (2020).

[38] L. Xue, J. Zhang, Y. Han, Phase separation induced ordered patterns in thin polymer blend films. Prog. Polym. Sci. 37, 564–594 (2012).

[39] J. Xu, Z. Liu, L. Jing, J. Chen, Fabrication of PCDTBT conductive network via phase separation. Materials 14, 5071 (2021). 34501162 10.3390/ma14175071 PMC8433801

[40] S. Hofle, T. Lutz, A. Egel, F. Nichel, S. W. Kettlitz, G. Gomard, U. Lemmer, A. Colsmann, Influence of the emission layer thickness on the optoelectronic properties of solution processed organic light-emitting diodes. ACS Photonics 1, 968–973 (2014).

