# Supplementary material for: Intrinsically stretchable three primary light-emitting films enabled by elastomer blend for polymer light-emitting diodes
Source: Sci Adv. 2023 Jun 21;9(25):eadh1504. doi: 10.1126/sciadv.adh1504 (PMC10284558; doi:10.1126/sciadv.adh1504)
Supplement: Supplementary file 1 — Figs. S1 to S53 Tables S1 to S3 Legend for movie S1 [file sciadv.adh1504_sm.pdf]

Science Advances
AAAS

Supplementary Materials for

Intrinsically stretchable three primary light-emitting films enabled by elastomer blend for polymer light-emitting diodes

Min Woo Jeong *et al.*

Corresponding author: Seong Jun Kang, junkang@khu.ac.kr, Jin Young Oh, jyoh@khu.ac.kr

Sci. Adv. **9**, eadh1504 (2023)
DOI: 10.1126/sciadv.adh1504

The PDF file includes:

Figs. S1 to S53
Tables S1 to S3
Legend for movie S1

Other Supplementary Material for this manuscript includes the following:

Movie S1

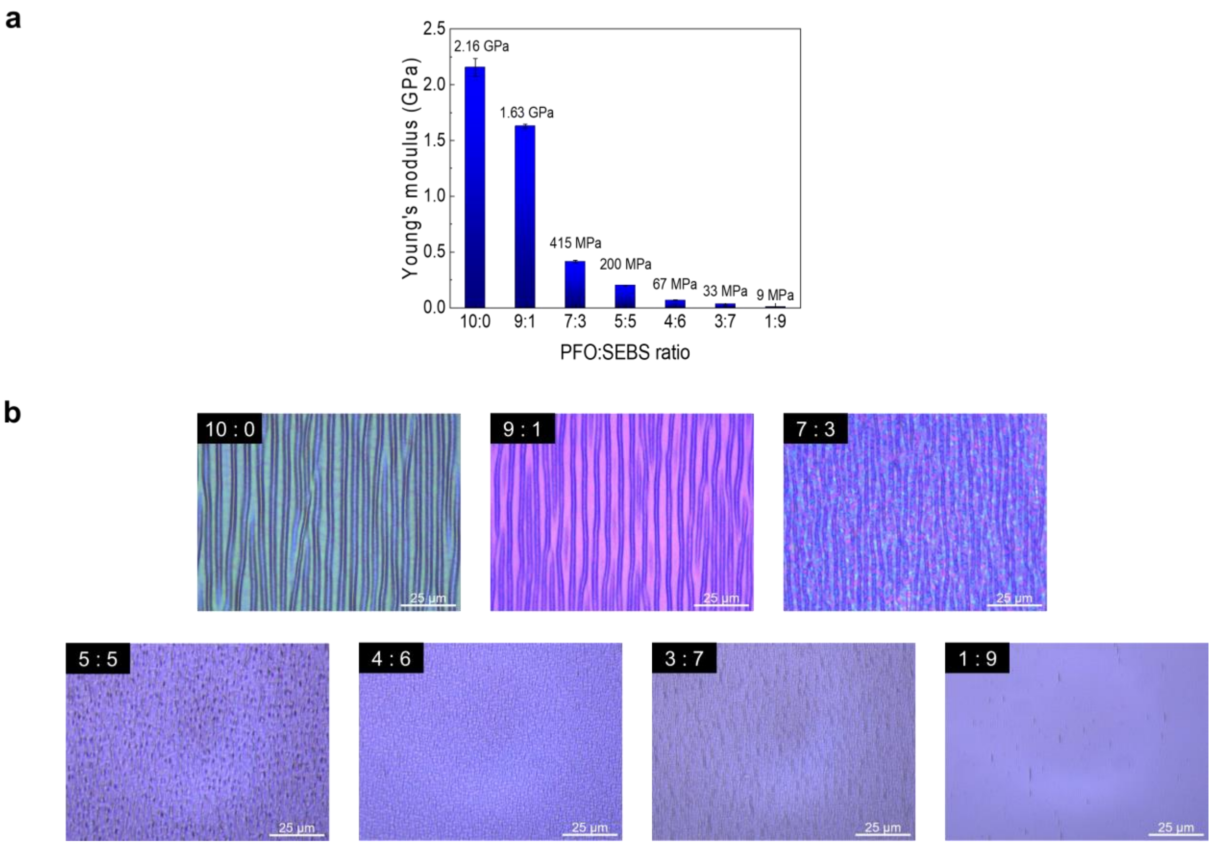

Fig. S1. Young's modulus of the light-emitting films. (a) Elastic modulus and (b) optical microscope images of PFO:SEBS blend films in various blend ratio are obtained using the wrinkle formation method.

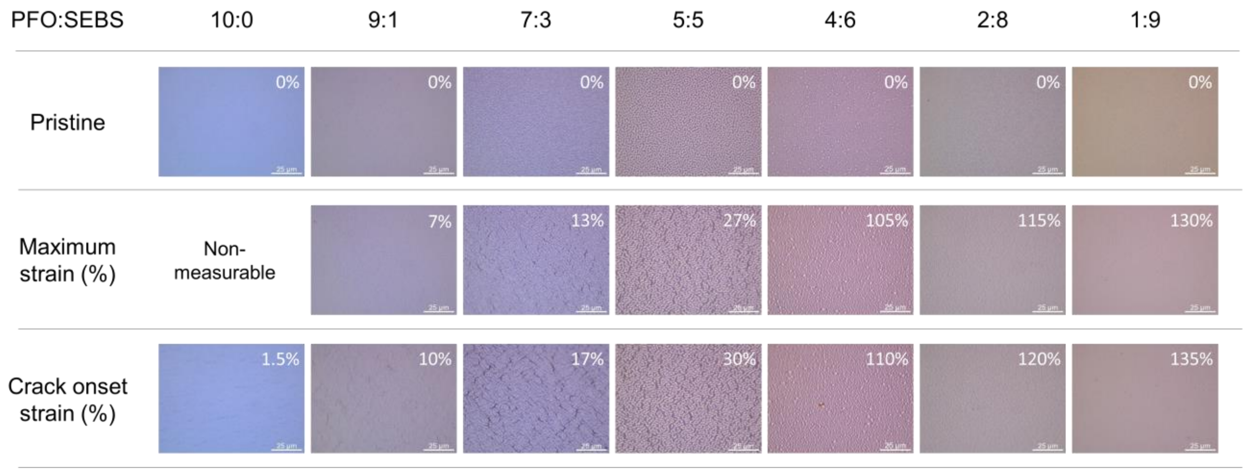

Fig. S2. Crack onset strains. Crack onset strains of PFO and SEBS blend films with similar thickness are observed using optical microscope and the images are separated three regions through initial (top), non-cracked maximum (middle), crack onset strain (bottom).

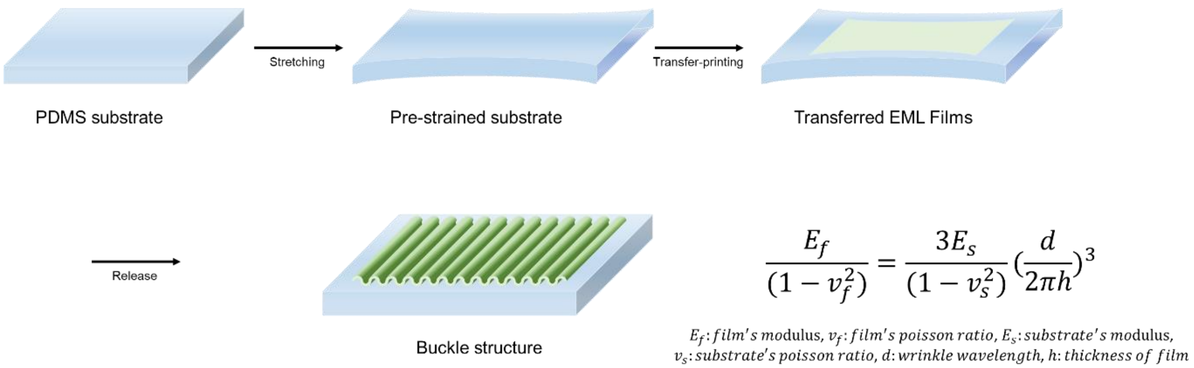

Fig. S3. Schematic illustration of elastic modulus calculation using wrinkle formation method.

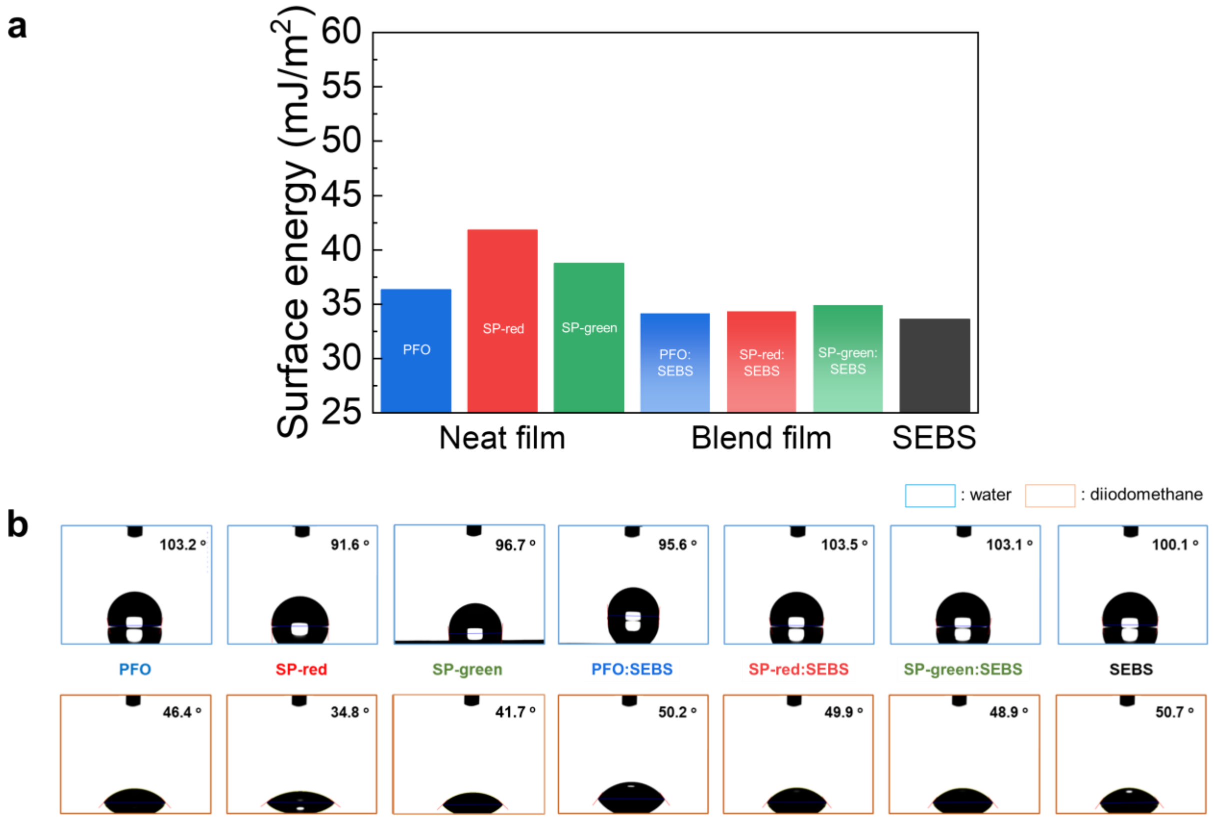

Fig. S4. Surface Energy and contact angle (CA) with DI-water and diiodomethane for each neat and blend red, green, and blue light-emitting films. (a) Surface energy was measured under the same conditions such as temperature, relative humidity and fluid's amount and also calculated by Owens-Wendt Method. (b) Contact angles of water and diiodomethane were suggested about neat RGB films and blend films.

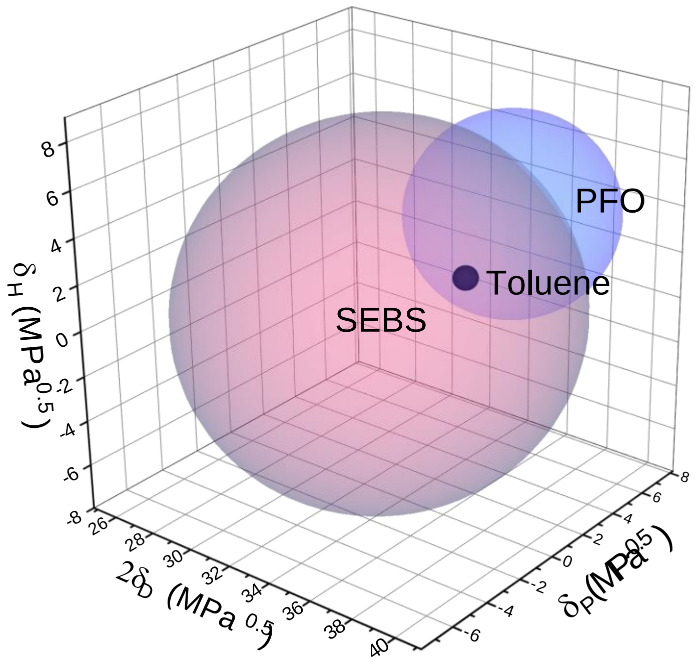

Fig. S5. The calculation of Hansen Solubility Spheres with solubility parameters. The Hansen Solubility Spheres were represented on 3D map plot for estimation of relative energy density (RED) value between PFO and SEBS. The spheres can be calculated with solubility parameters, which are dispersive solubility parameter (δ_D), polar solubility parameter (δ_P), hydrogen bonding parameter (δ_H), total Hansen solubility parameter (δ_r) and sphere radius (R_0). PFO and SEBS's solubility parameters are 18.55, 2.8, 4.51, 19.81, 4.1 Mpa$^{1/2}$ and 16.388, 0.198, 0, 16.39, 8 Mpa$^{1/2}$, respectively.

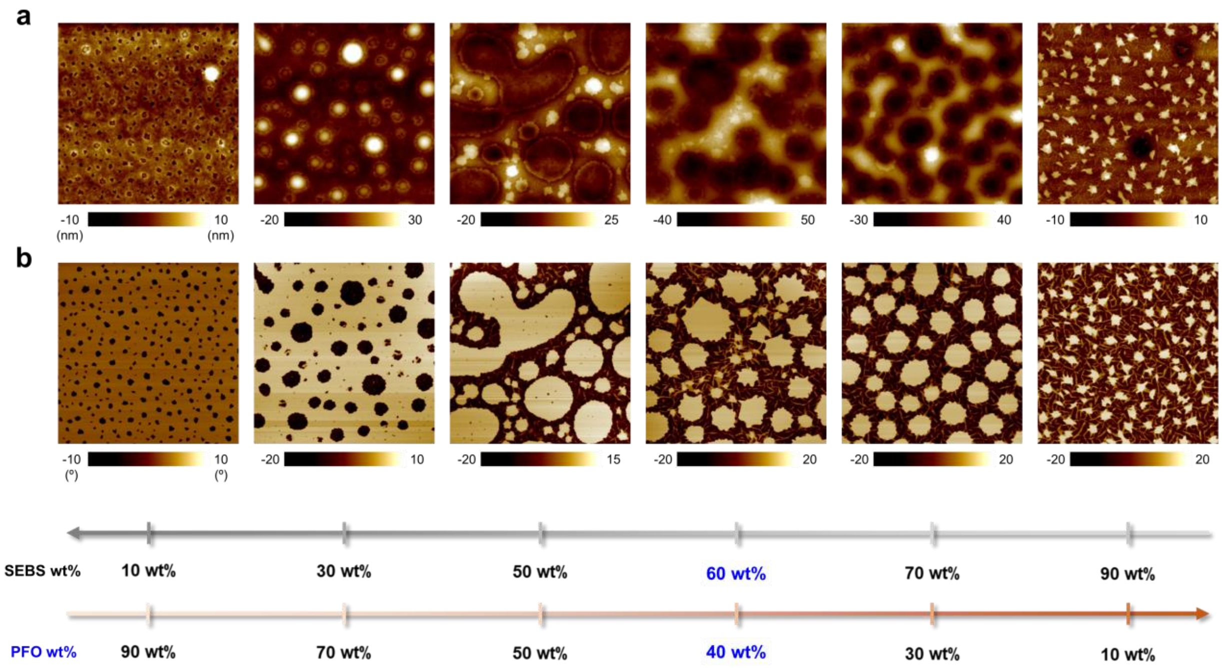

Fig. S6. Nanomorphology analysis of the blend films. The AFM (a) height and (b) phase images show the morphology changes of phase separation depended on the blend ratio of PFO:SEBS films. The blend films were fabricated with the same thickness and concentration for better observation of morphology changes.

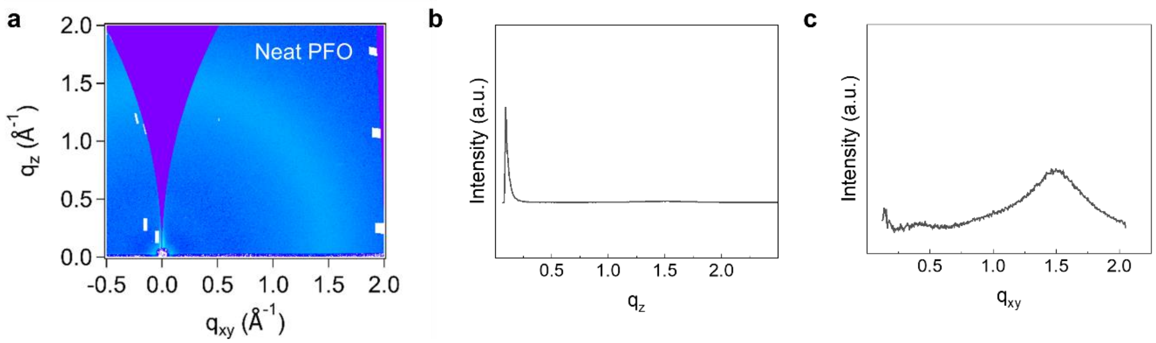

Fig. S7. 2D GIWAX analysis of neat PFO film. (a) The two-dimensional diffraction pattern images by GIWAX analysis and one-dimensional curves with (b) out of plane and (c) in plane both directions.

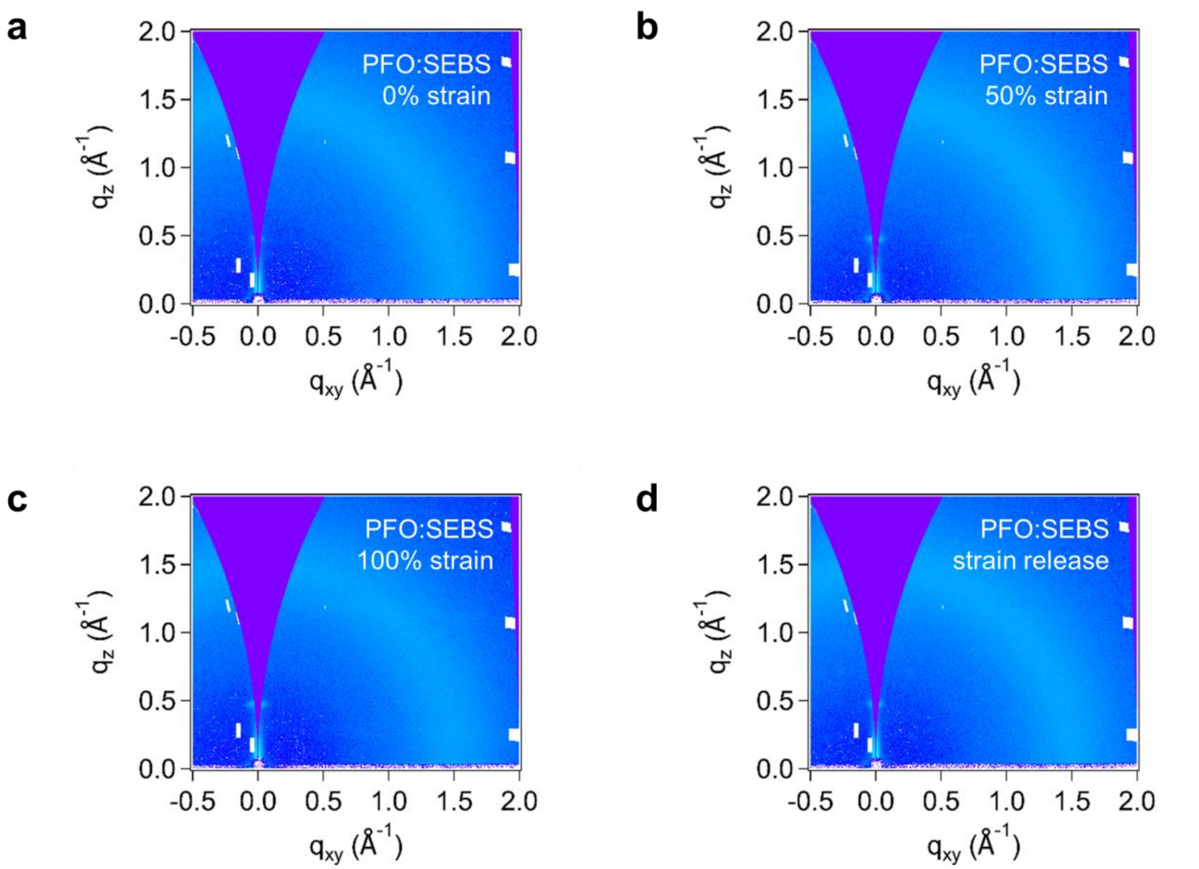

Fig. S8. 2D GIWAX patterns of PFO:SEBS 4:6 blend films. Two-dimensional diffraction pattern images by GIWAX analysis on various strain: (a) 0%, (b) 50%, (c) 100% strained and (d) strain released PFO:SEBS blend films.

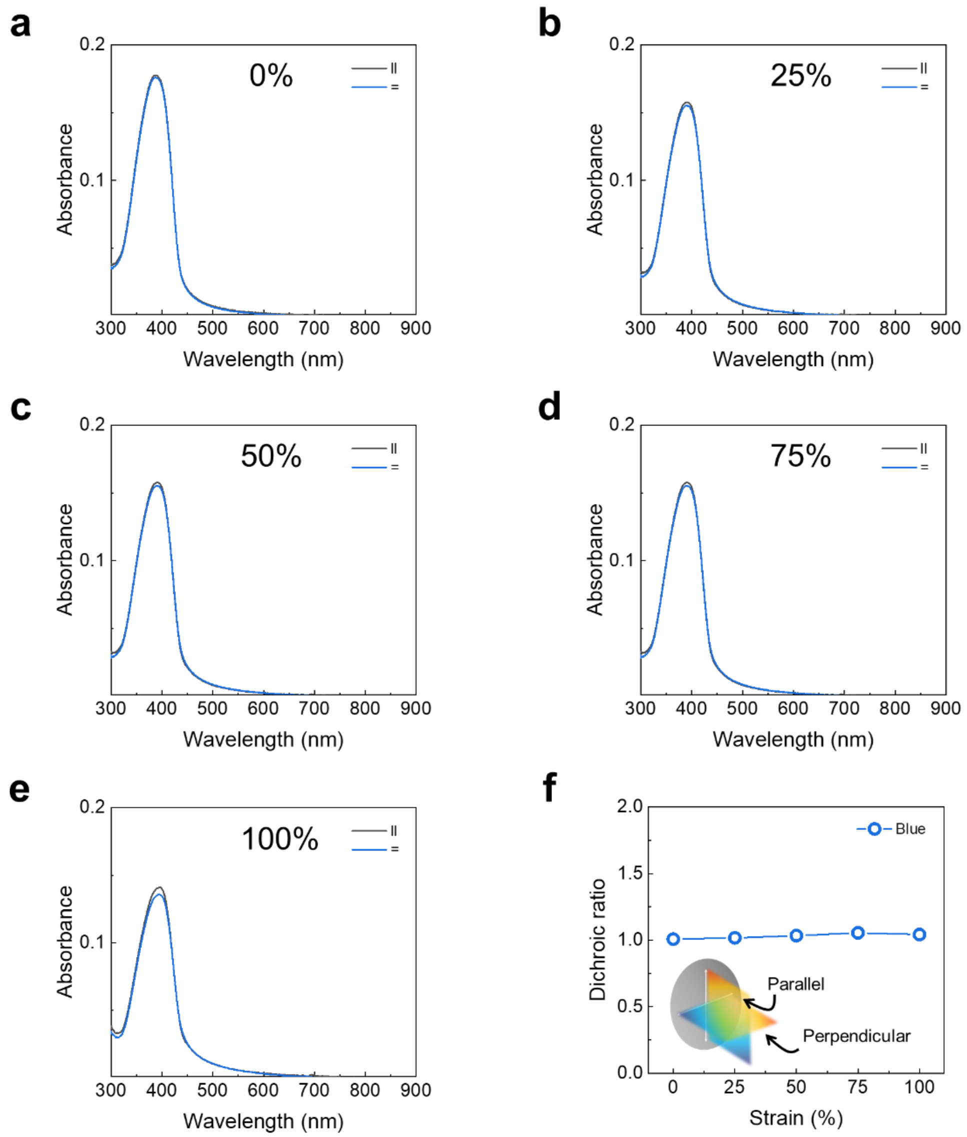

Fig. S9. Polarized UV-vis spectroscopy of PFO:SEBS 4:6 blend films under different strains. PFO:SEBS films are transferred to bare glass substrate after stretching and analyzed absorbance intensity of (a) 0%, (b) 25%, (c) 50%, (d) 75%, (e) 100% strain. (f) The dichroic ratios are determined by $I_{parellel}/I_{perpendicular}$.

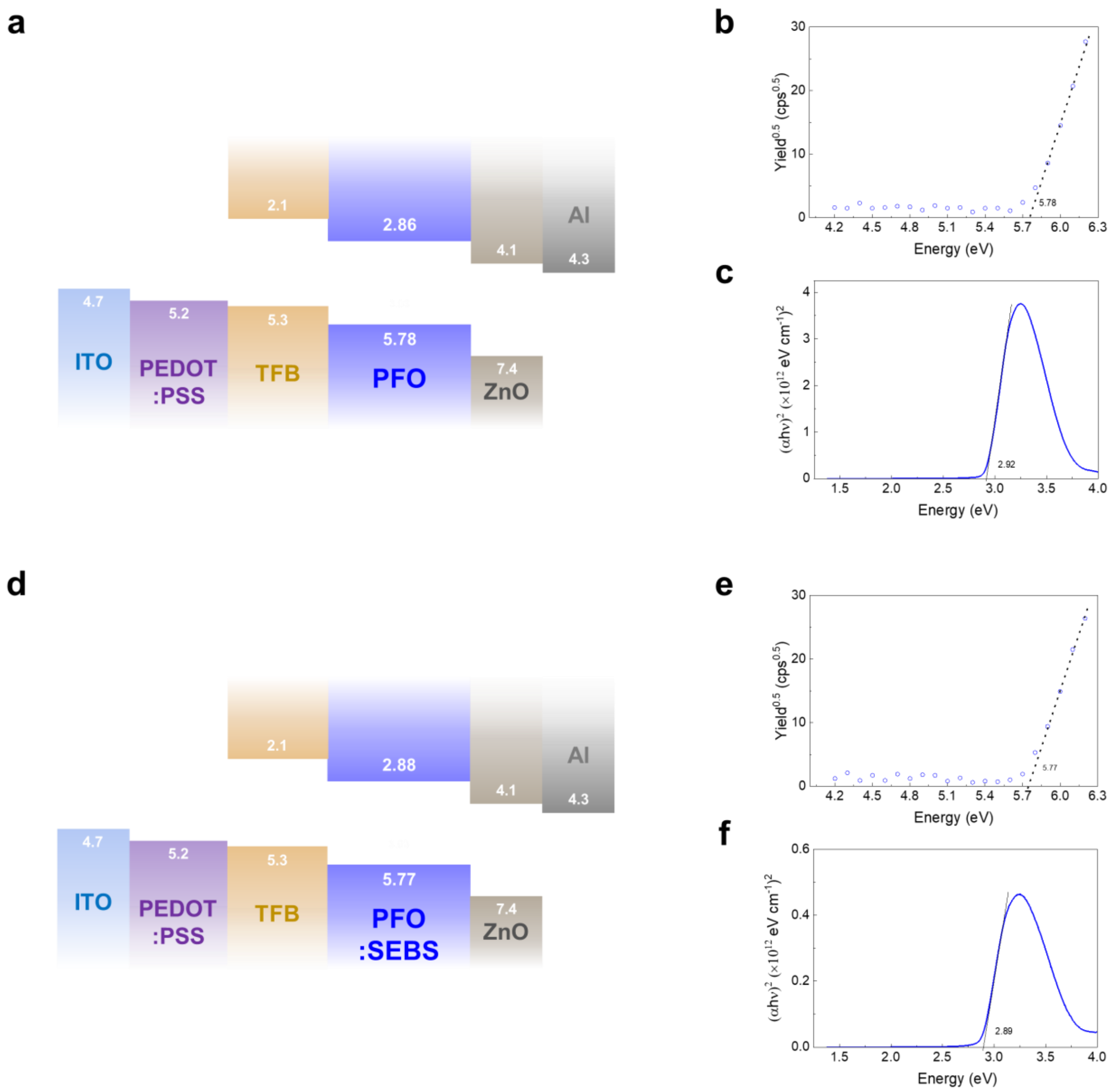

Fig. S10. Energy-band alignment of blue PLEDs. (a,d) Energy-band diagram of all components in blue-PLED devices, (b,e) PESA analysis to determine (b) PFO and (e) PFO:SEBS film's HOMO level, (c,f) Tauc' plot results by UV-vis spectroscopy with each (c) PFO and (f) PFO:SEBS films.

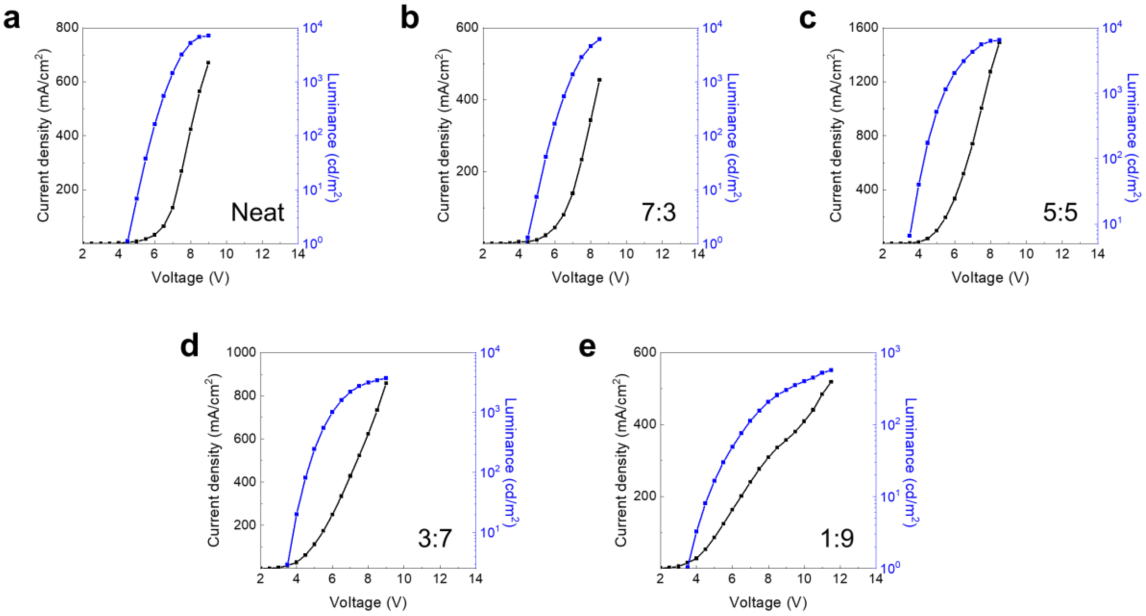

Fig. S11. Current density-voltage-luminance (J-V-L) characteristics of PLED devices with ITO/PEDOT:PSS/TFB/PFO:SEBS/ZnO/Al device structure. The PLED devices using PFO:SEBS blend films showed the electroluminescence performance with (a) neat PFO, (b) 7:3, (c) 5:5, (d) 3:7, (e) 1:9 (PFO:SEBS) blend ratios.

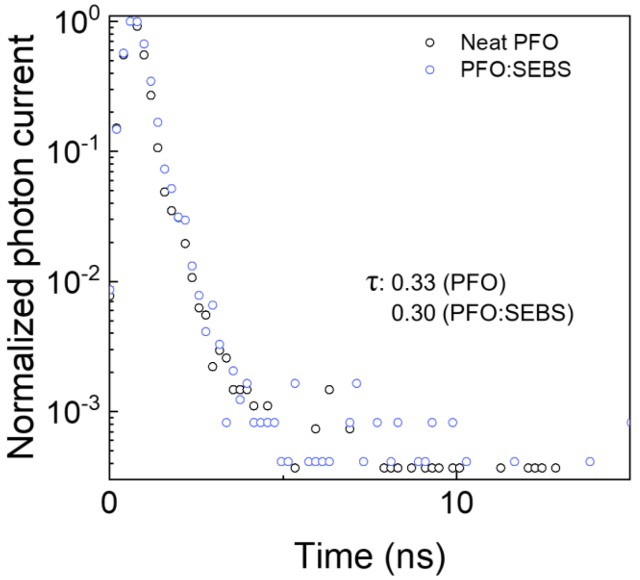

Fig. S12. Time-resolved photoluminescence (TRPL) spectra of neat and blended light-emitting layers. The photon current is obtained by time-flow with blue light emitting films on quartz substrate. The samples were excited using 365nm wavelength pulsed laser.

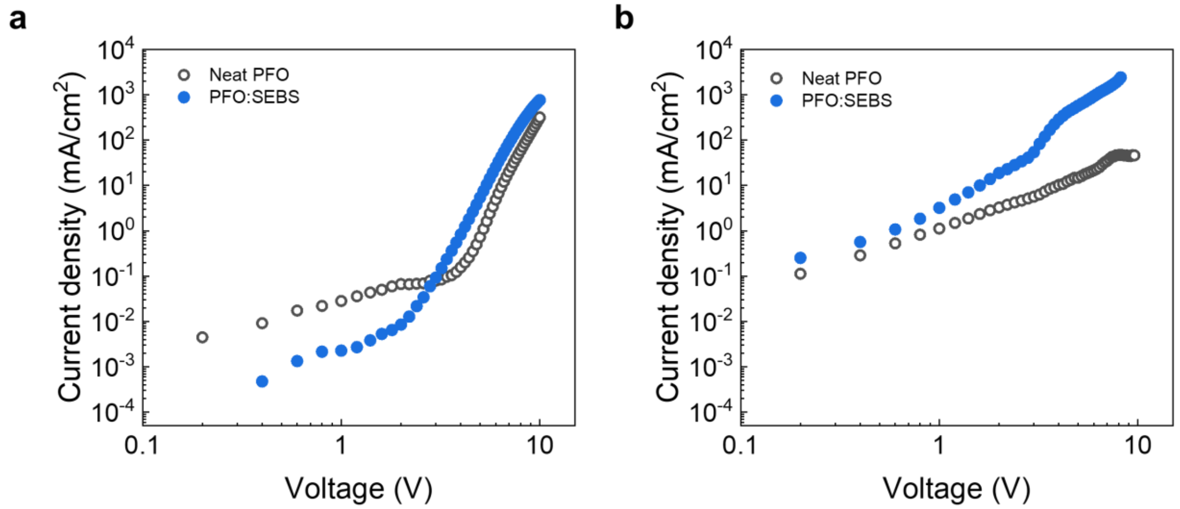

Fig. S13. Hole only and electron only devices (HOD and EOD) for evaluation of charge carrier transport property. Each current density - voltage curve was measured with (a) HOD and (b) EOD devices with neat PFO and PFO:SEBS blend film, respectively.

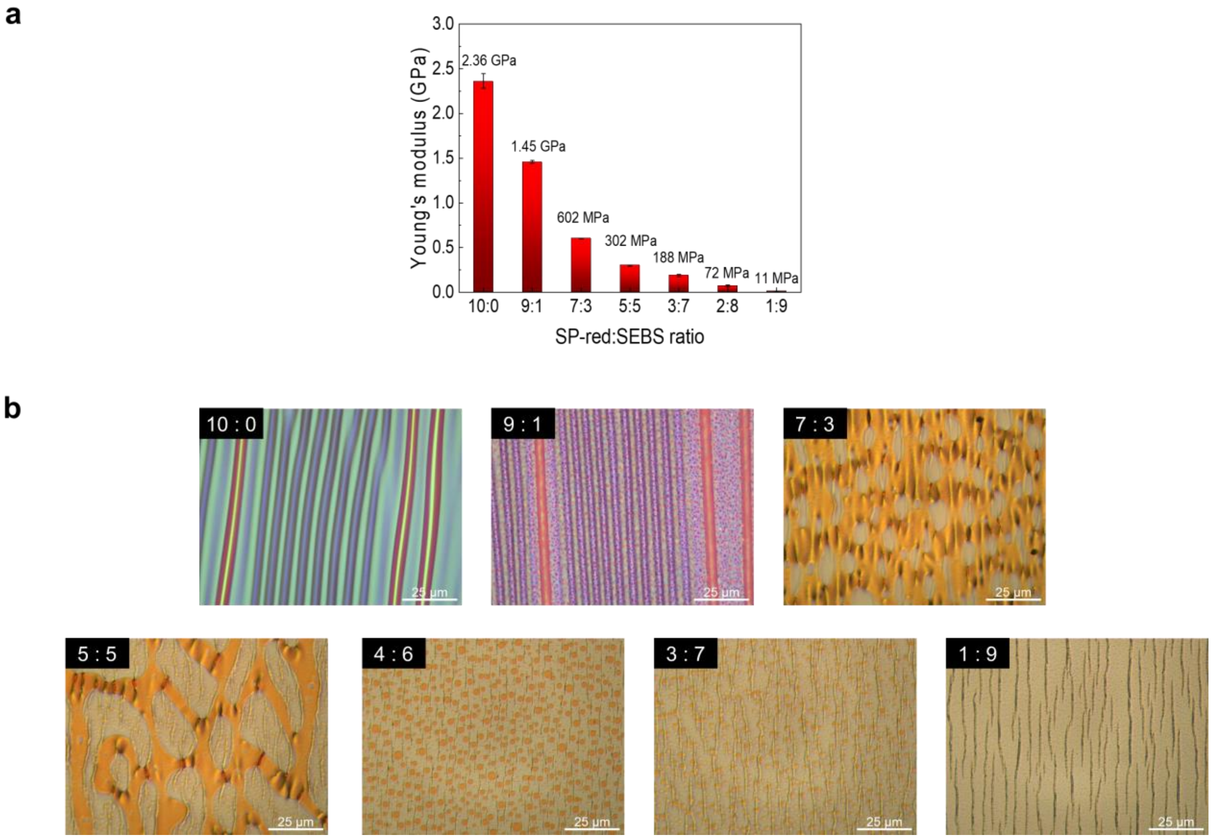

Fig. S14. Young's modulus of the light-emitting films. (a) Elastic modulus and (b) optical microscope images of SP-red:SEBS blend films in various blend ratio are obtained using the wrinkle formation method.

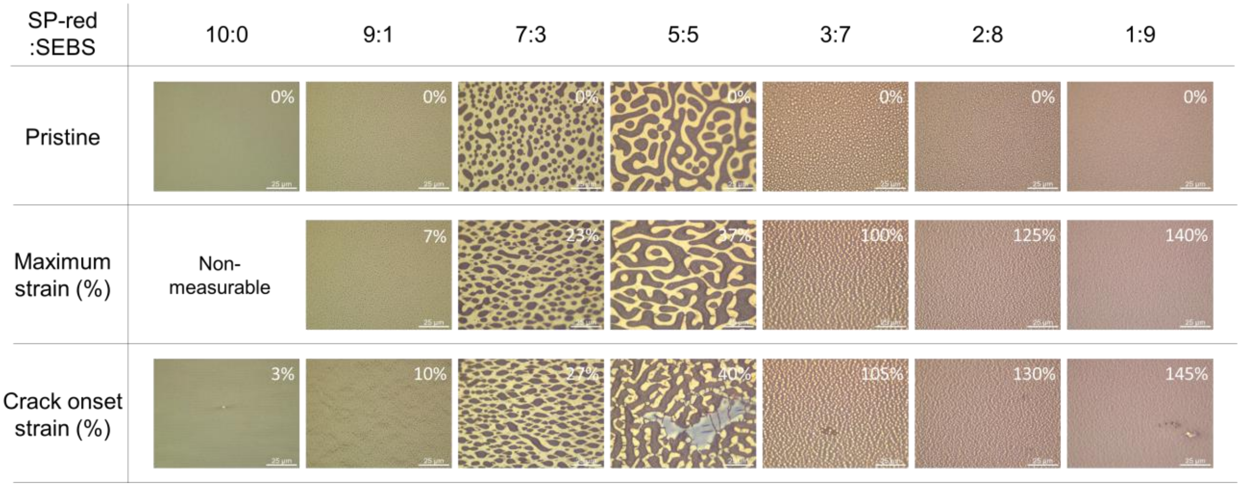

Fig. S15. Crack onset strains. Crack onset strains of SP-red and SEBS blend films with similar thickness are observed using optical microscope and the images are separated three regions through initial (top), non-cracked maximum (middle), crack onset strain (bottom).

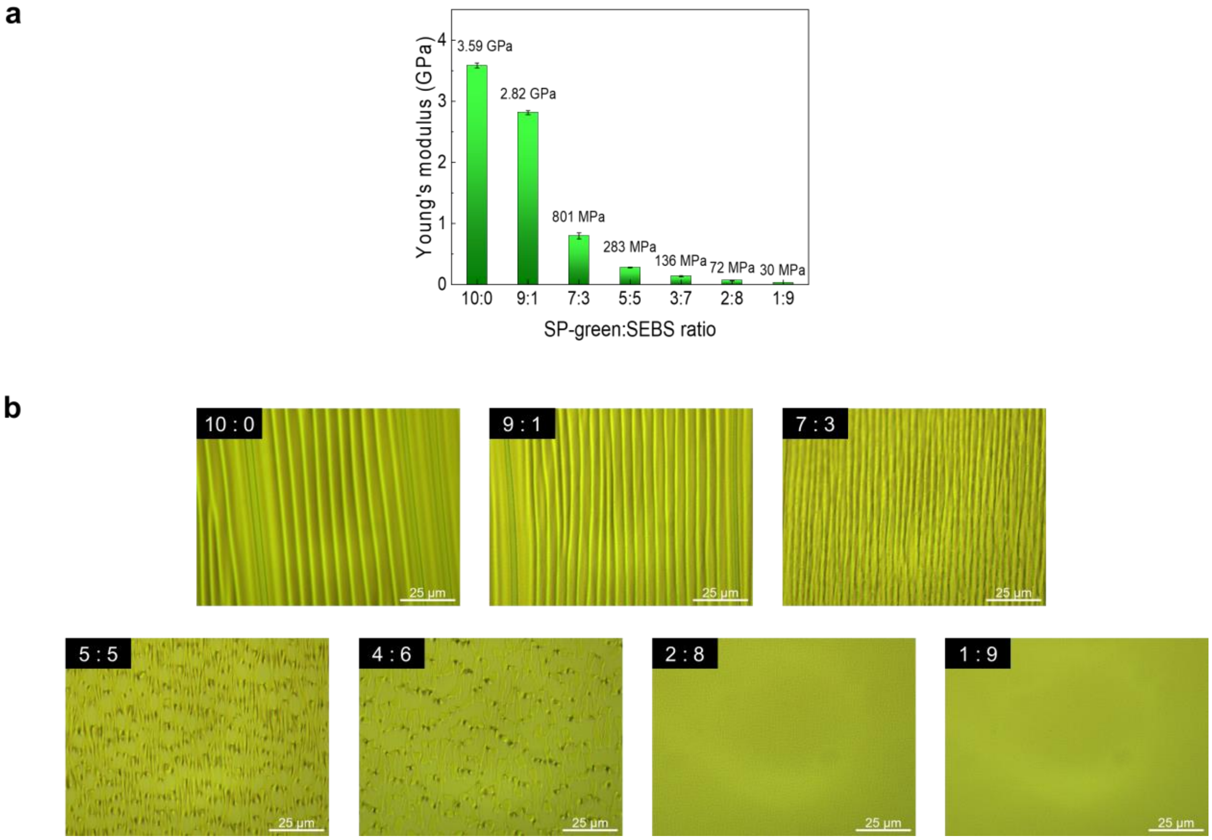

Fig. S16. Young's modulus of the light-emitting films. (a) Elastic modulus and (b) optical microscope images of SP-green:SEBS blend films in various blend ratios are obtained using the wrinkle formation method.

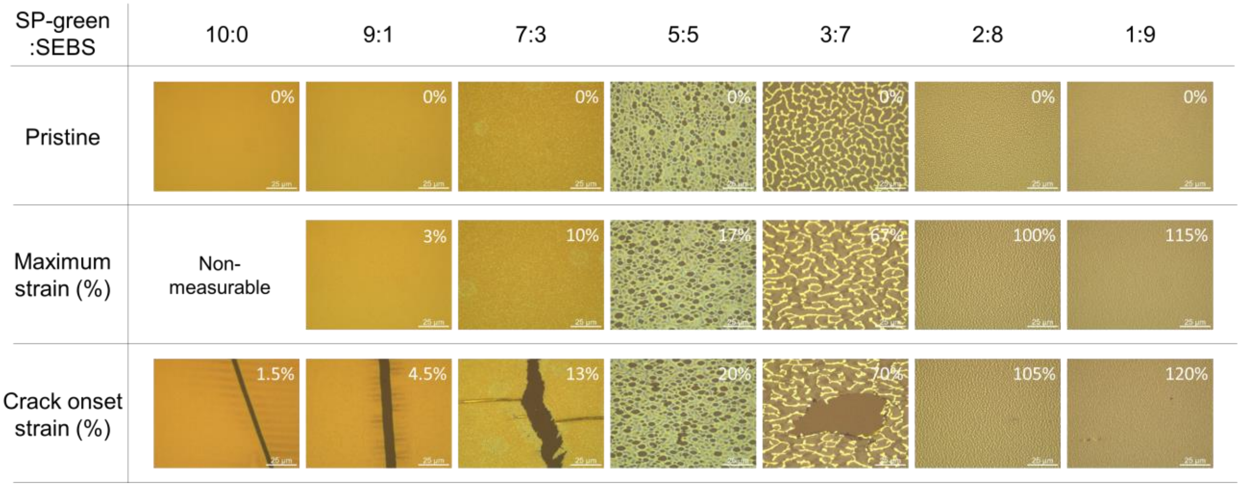

Fig. S17. Crack onset strains. Crack onset strains of SP-green and SEBS blend films with similar thickness are observed using optical microscope and the images are separated three regions through initial (top), non-cracked maximum (middle), crack onset strain (bottom).

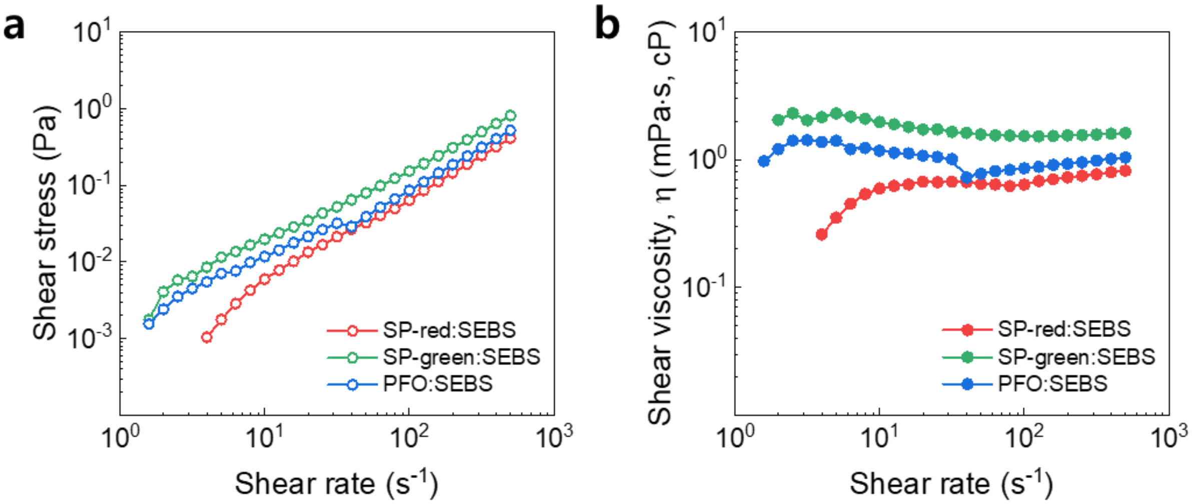

$$Shear\ viscosity\ (mPa \cdot s) = \frac{Shear\ stress\ (mPa)}{Shear\ rate\ (\frac{1}{s})}$$

Fig. S18. Shear stress and shear viscosity of optimized three primary color light-emitting blending solutions. (a) Shear stress and (b) shear viscosity (η) change of optimized R,G,B light-emitting polymer:SEBS blend solutions as a function of shear rate.

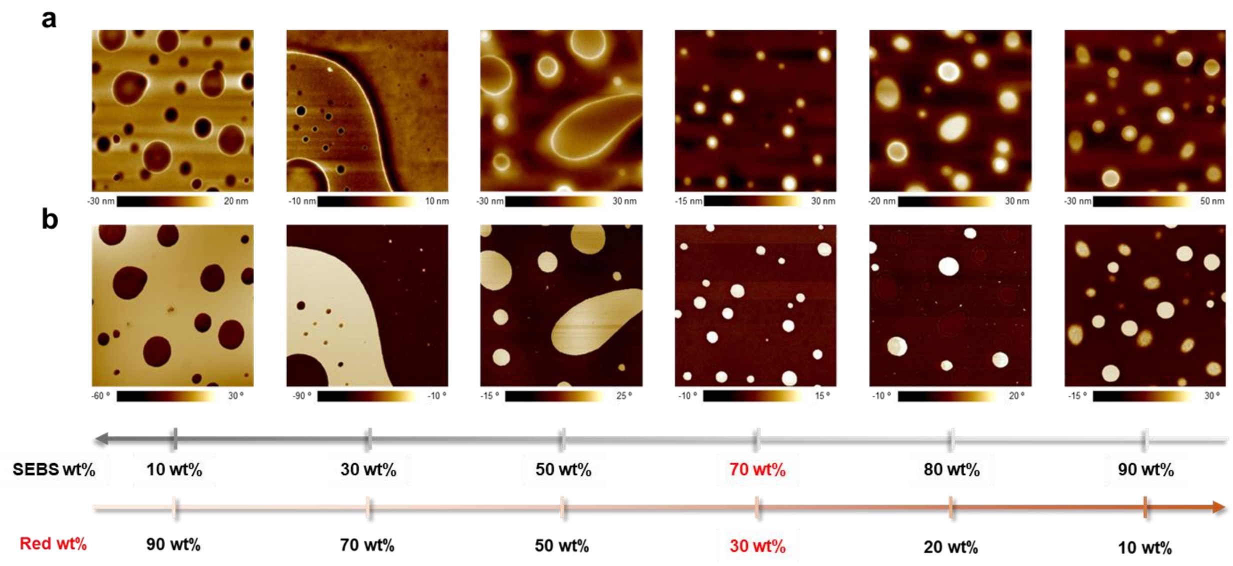

Fig. S19. Nanomorphology analysis of the blend films. The AFM (a) height and (b) phase images show the morphology changes of phase separation depended on the blend ratio of SP-red:SEBS films. The blend films were fabricated with the same thickness and concentration for better observation of morphology changes.

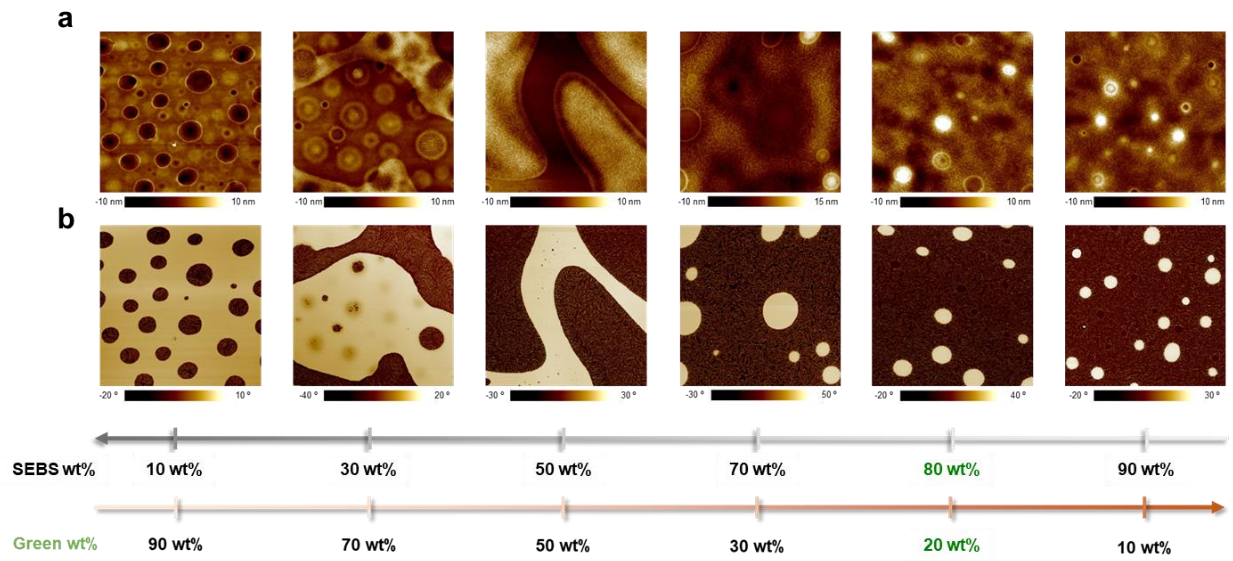

Fig. S20. Nanomorphology analysis of the blend films. The AFM (a) height and (b) phase images show the morphology changes of phase separation depended on the blend ratio of SP-green:SEBS films. The blend films were fabricated with the same thickness and concentration for better observation of morphology changes.

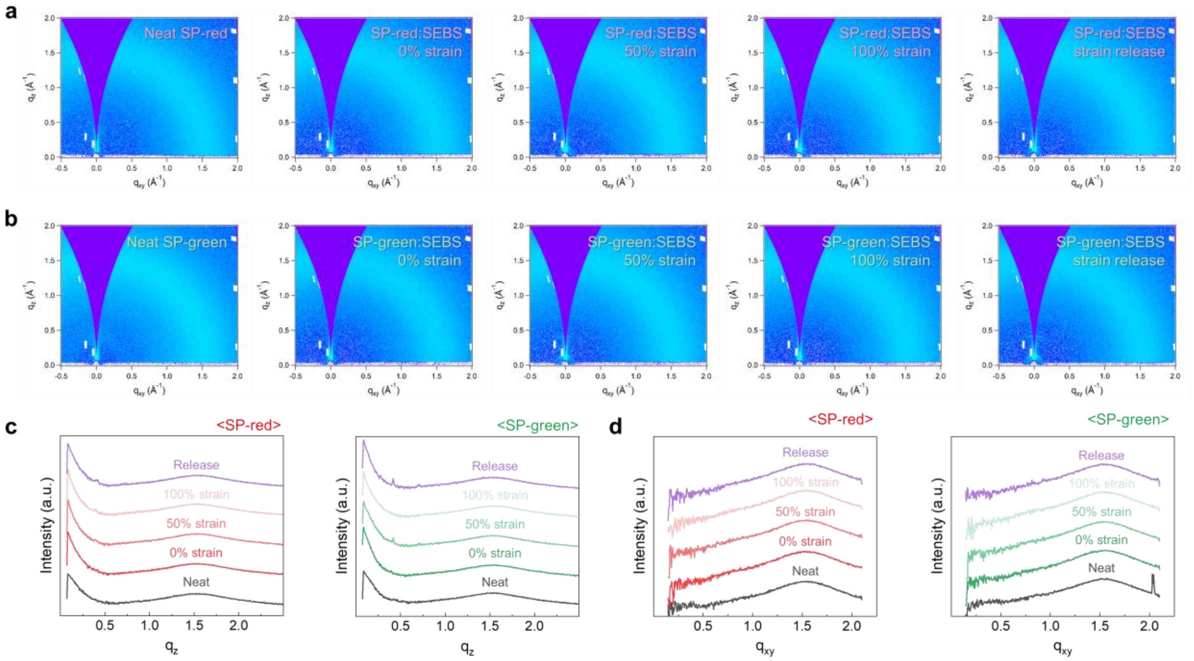

Fig. S21. GIWAX analysis of SP-red and SP-green light emitting polymer blend films. GIWAX 2D diffraction images of (a) SP-red:SEBS and (b) SP-green:SEBS blend films and their 1D curves for (c) out-of plane and (d) in plane.

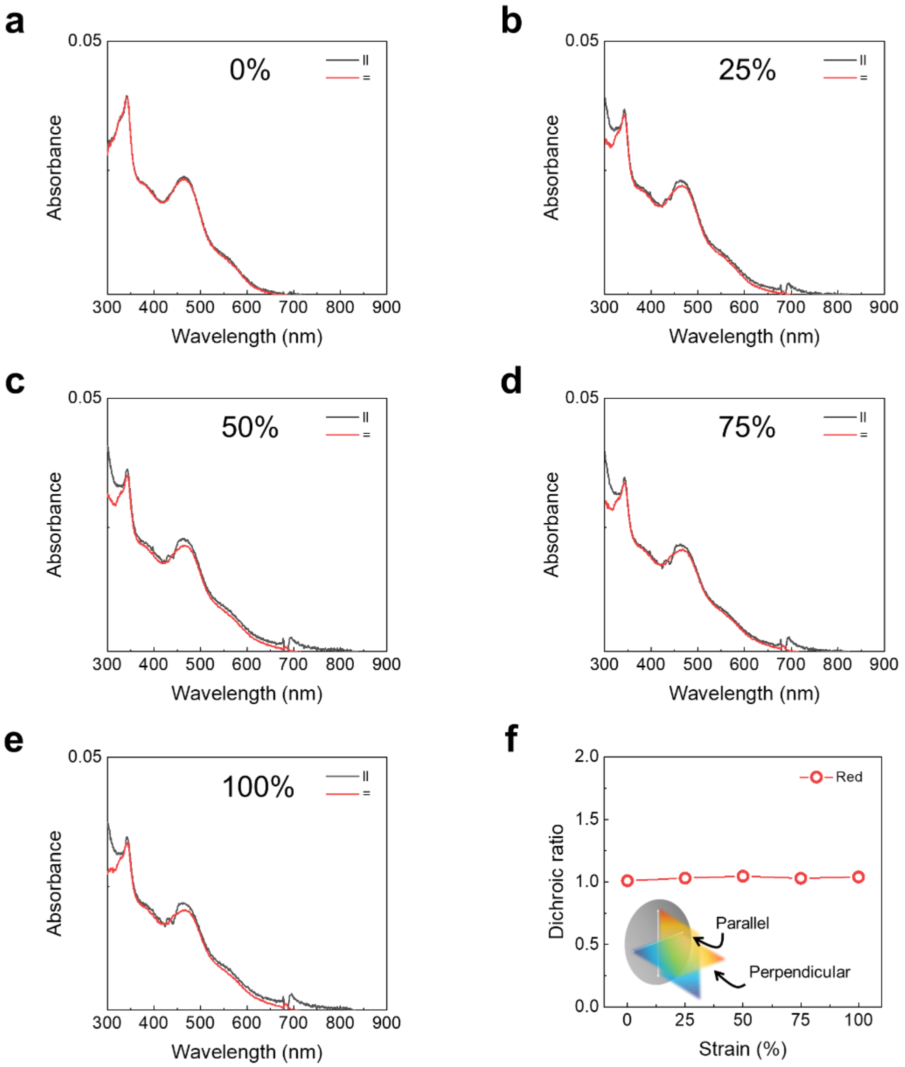

Fig. S22. Polarized UV-vis spectroscopy of SP-red:SEBS 4:6 blend films under different strains. SP-red:SEBS films are transferred to bare glass substrate after stretching and analyzed absorbance intensity of (a) 0%, (b) 25%, (c) 50%, (d) 75%, (e) 100% strain. (f) The dichroic ratios are determined by $I_{parellel}/I_{perpendicular}$.

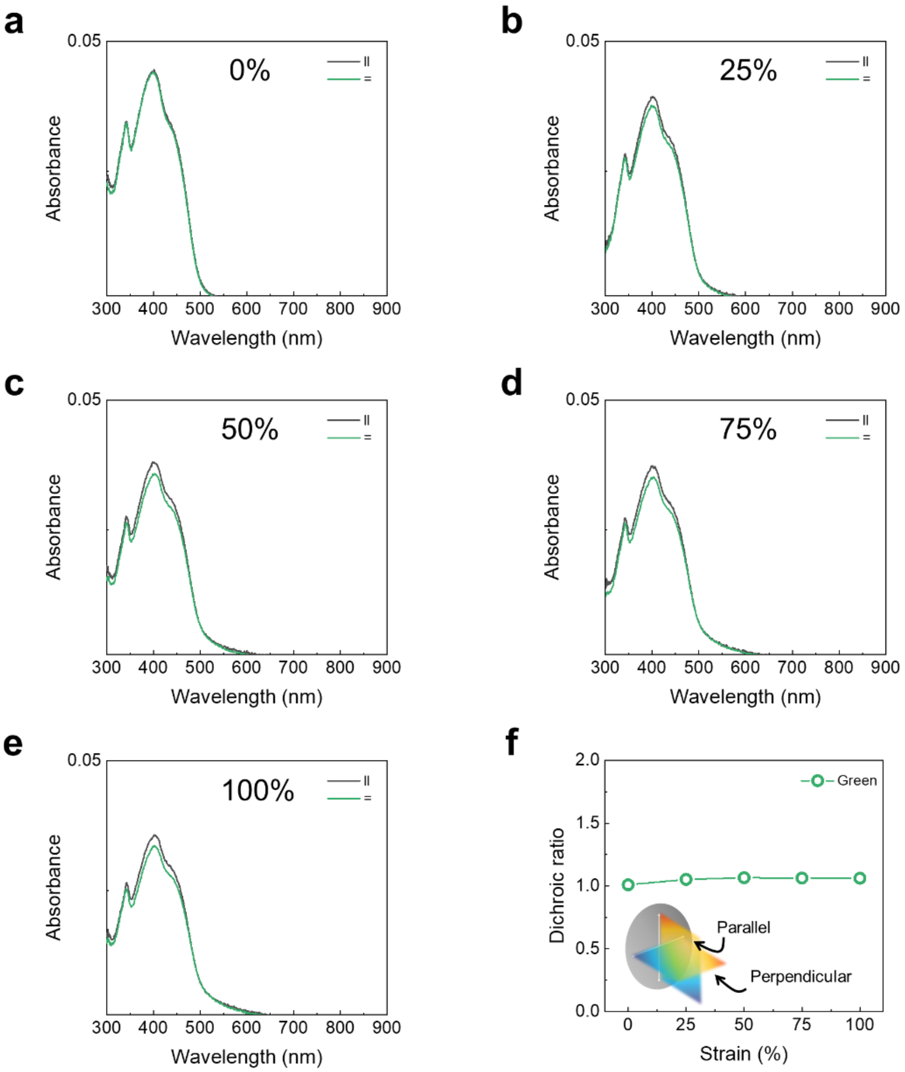

Fig. S23. Polarized UV-vis spectroscopy of SP-green:SEBS 4:6 blend films under different strains. SP-green:SEBS films are transferred to bare glass substrate after stretching and analyzed absorbance intensity of (a) 0%, (b) 25%, (c) 50%, (d) 75%, (e) 100% strain. (f) The dichroic ratios are determined by $I_{parellel}/I_{perpendicular}$.

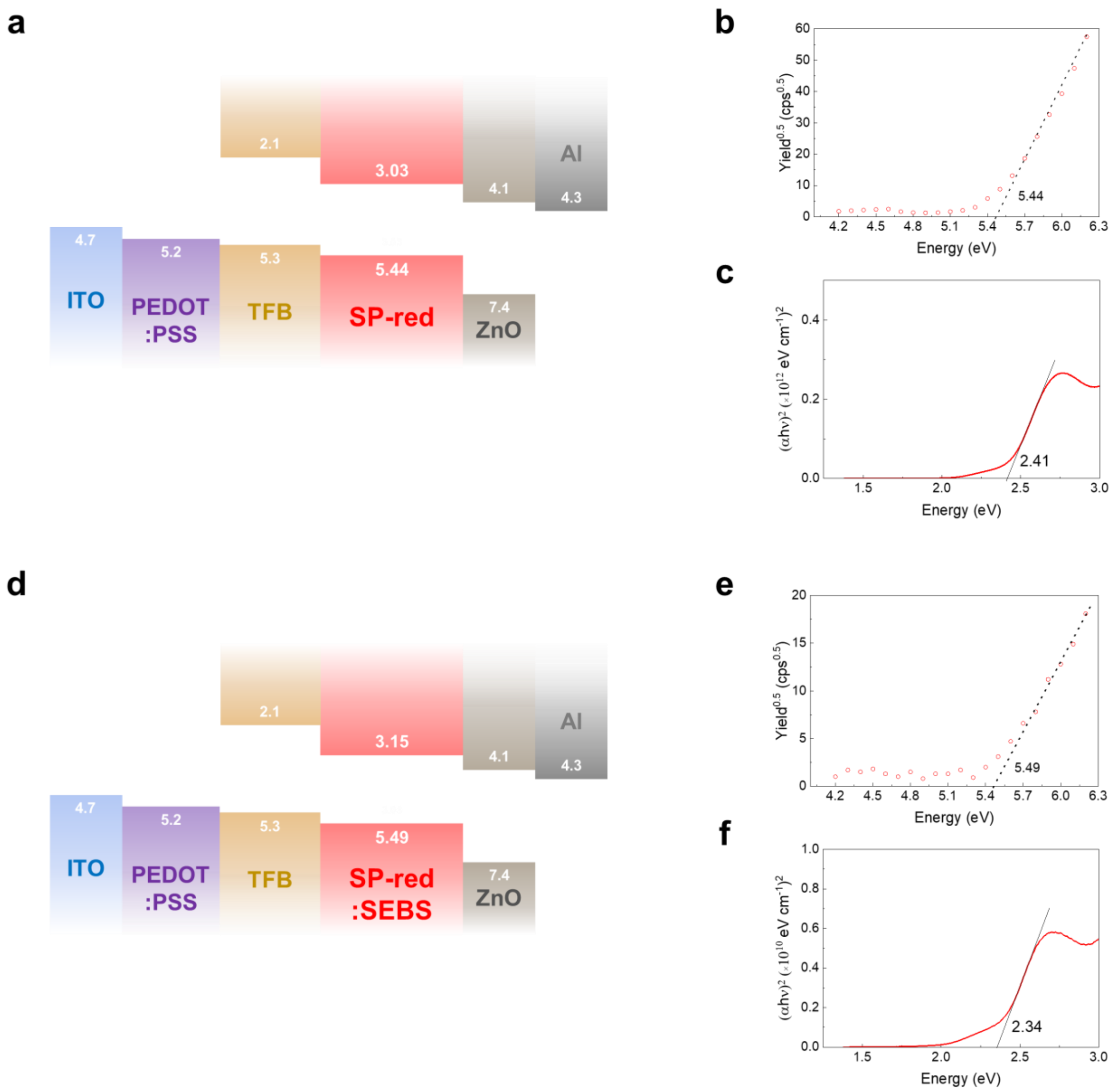

Fig. S24. Energy-band alignment of red-PLEDs. (a,d) Energy-band diagram of all components in red-PLED devices, (b,e) PESA analysis to determine (b) SP-red and (e) SP-red:SEBS film's HOMO level, (c,f) Tauc' plot results by UV-vis spectroscopy with each (c) SP-red and (f) SP-red:SEBS films.

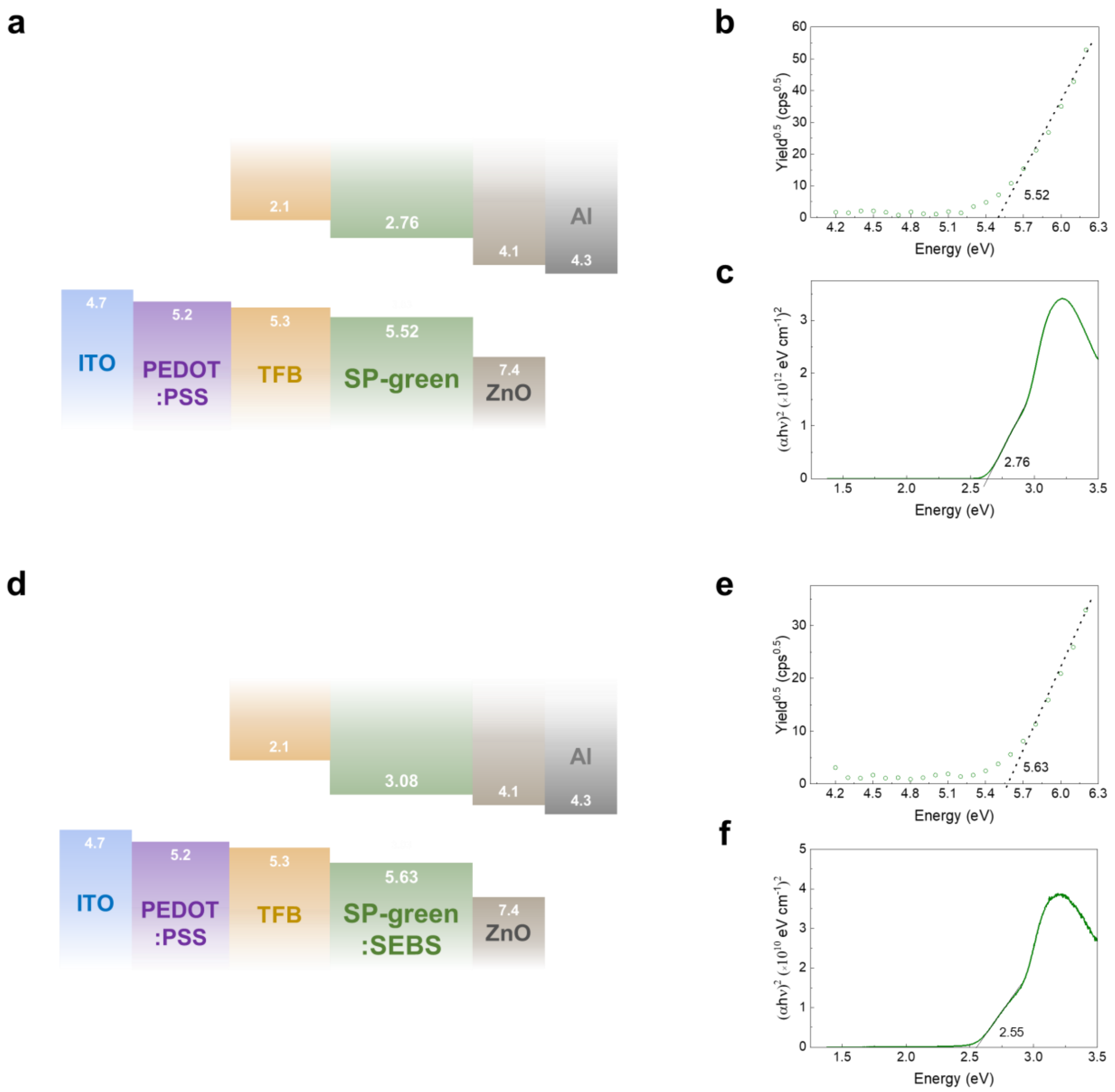

Fig. S25. Energy-band alignment of green-PLEDs. (a,d) Energy-band diagram of all components in green-PLED devices, (b,e) PESA analysis to determine (b) SP-green and (e) SP-green:SEBS film's HOMO level, (c,f) Tauc' plot results by UV-vis spectroscopy with each (c) SP-green and (f) SP-green:SEBS films.

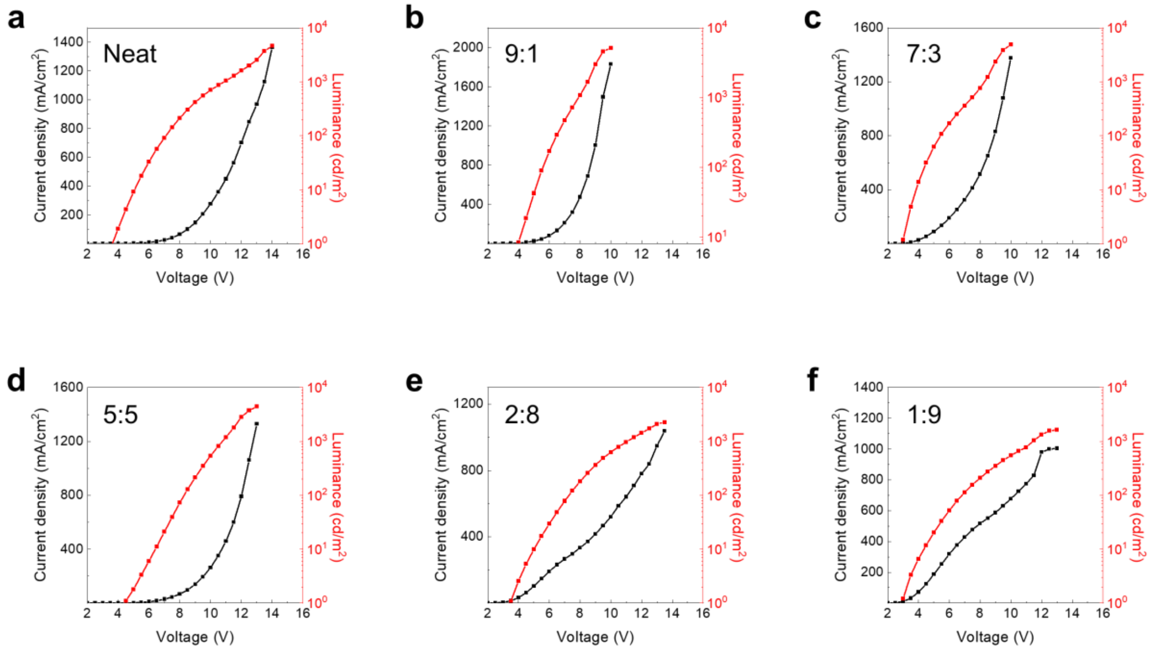

Fig. S26. Current density-voltage-luminance (*J*-*V*-*L*) characteristics of PLED devices with ITO/PEDOT:PSS/TFB/SP-red:SEBS/ZnO/Al device structure. The PLED devices using SP-red:SEBS blend films showed the electroluminescence performance with (a) neat SP-red, (b) 9:1, (c) 7:3, (d) 5:5, (e) 2:8, and (f) 1:9 (SP-red:SEBS) blend ratios.

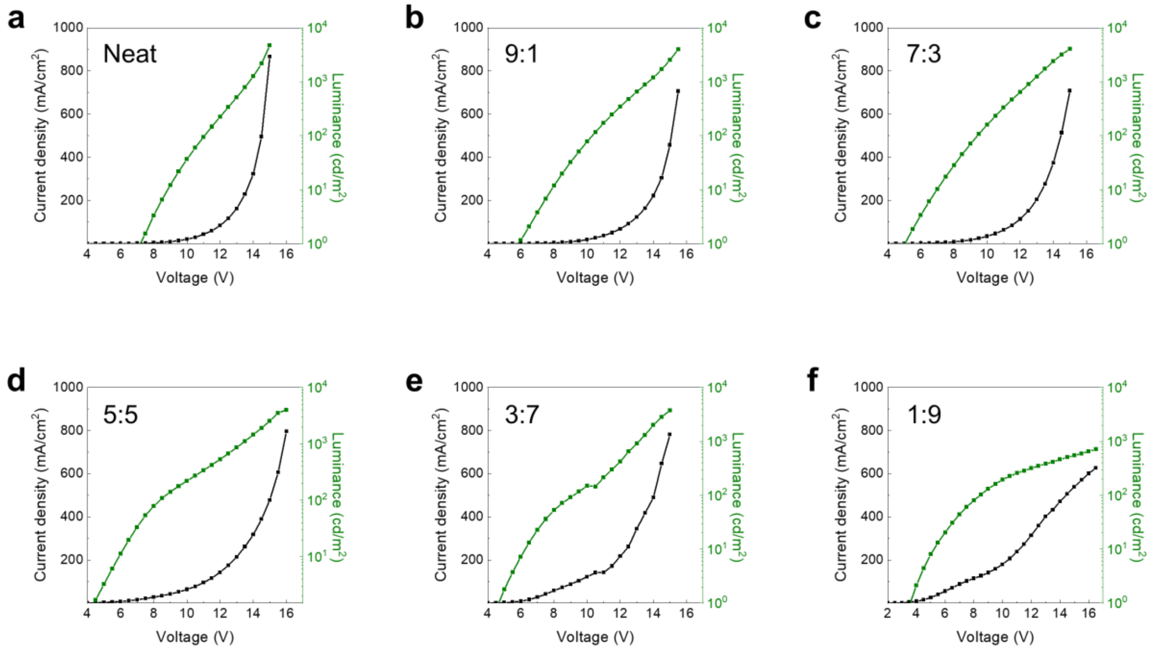

Fig. S27. Current density-voltage-luminance (*J*-*V*-*L*) characteristics of PLED devices with ITO/PEDOT:PSS/TFB/SP-green:SEBS/ZnO/Al device structure. The PLED devices using SP-green:SEBS blend films showed the electroluminescence performance with (a) neat SP-green, (b) 9:1, (c) 7:3, (d) 5:5, (e) 3:7, and (f) 1:9 (SP-green:SEBS) blend ratios.

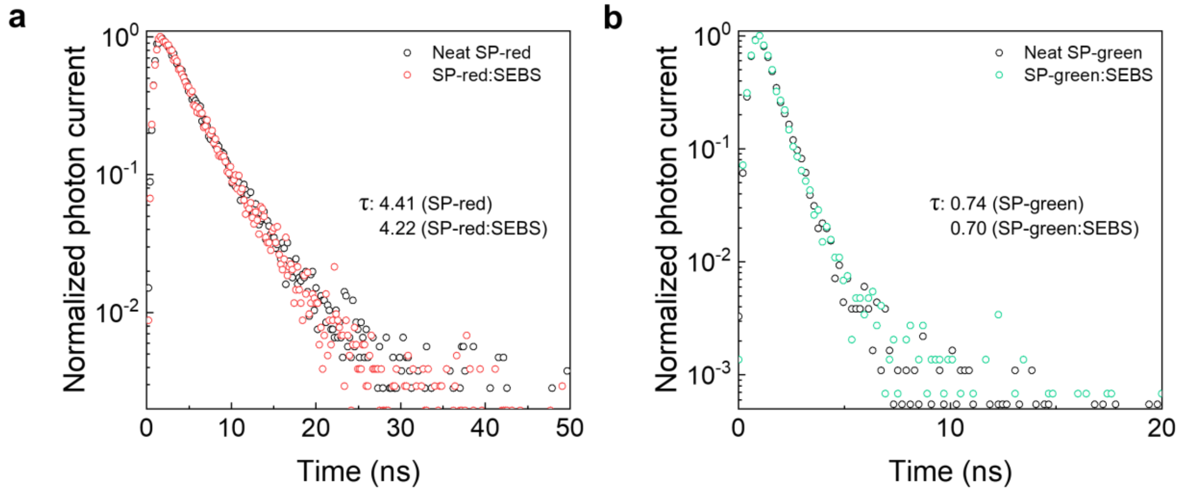

Fig. S28. Time-resolved photoluminescence (TRPL) spectra of neat and blended light-emitting layers for comparing average lifetime. The photon current is obtained by time-flow with (a) red and (b) green light emitting films on quartz substrate. The samples were excited using 470nm and 405nm wavelength pulsed lasers, respectively.

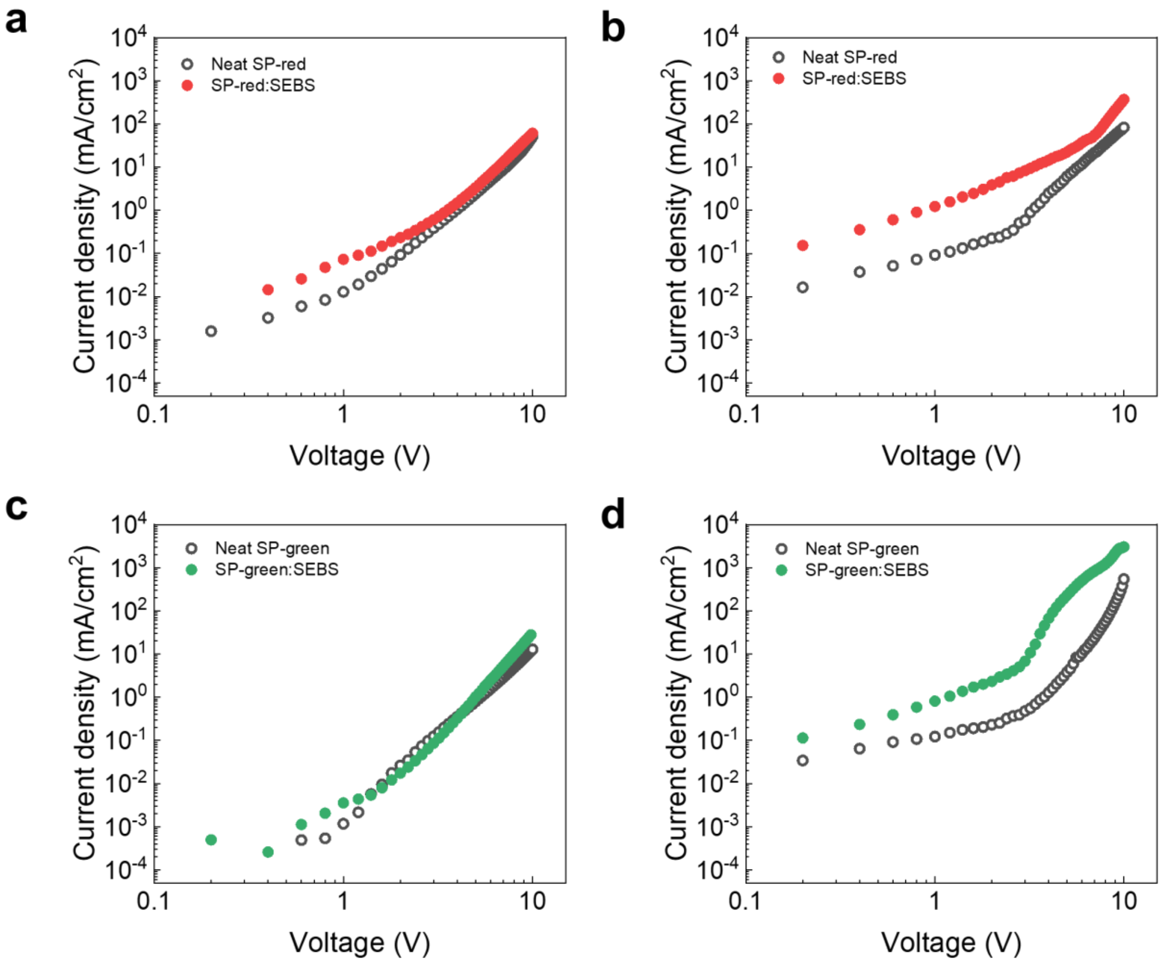

Fig. S29. Hole only and electron only devices (HOD and EOD) for evaluation of charge carrier transport property. Each current density - voltage curve was measured with (a) HOD and (b) EOD devices using neat SP-red and SEBS blend films EML and also measured with neat SP-green and SEBS blend film EML's (c) HOD and (d) EOD devices, respectively.

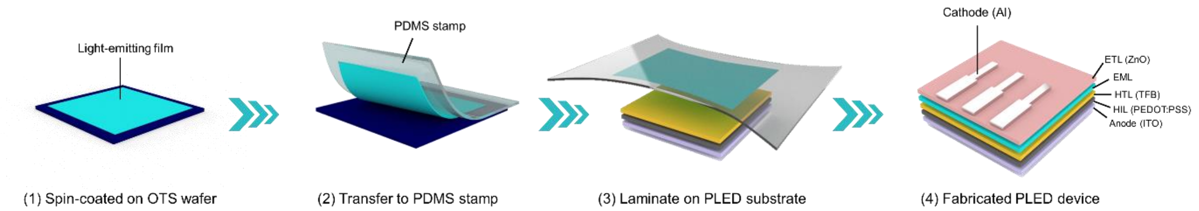

Fig. S30. Schematic illustration of transfer printing method of light-emitting blend films. The blend films are transferred with PDMS stamp from OTS treated SiO_2 silicon wafer to PLED substrates.

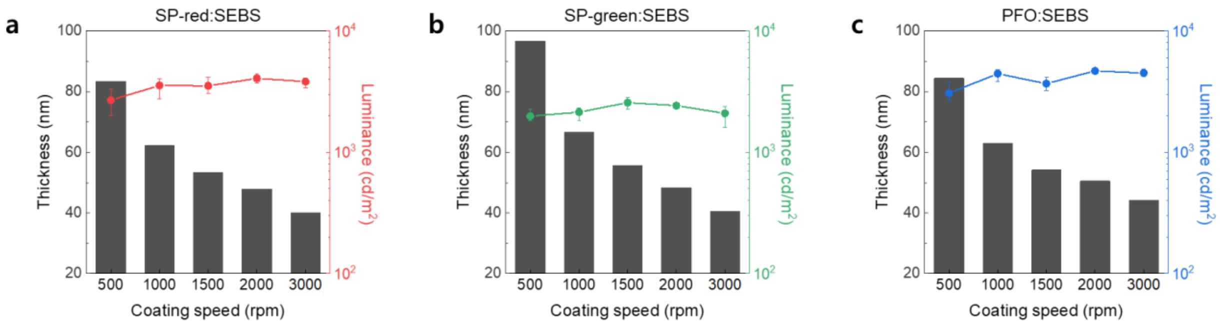

Fig. S31. Film thickness and luminance as function of spin-coating speed. (a)SP-red:SEBS, (b)SP-green:SEBS, and (c)PFO:SEBS blend solutions.

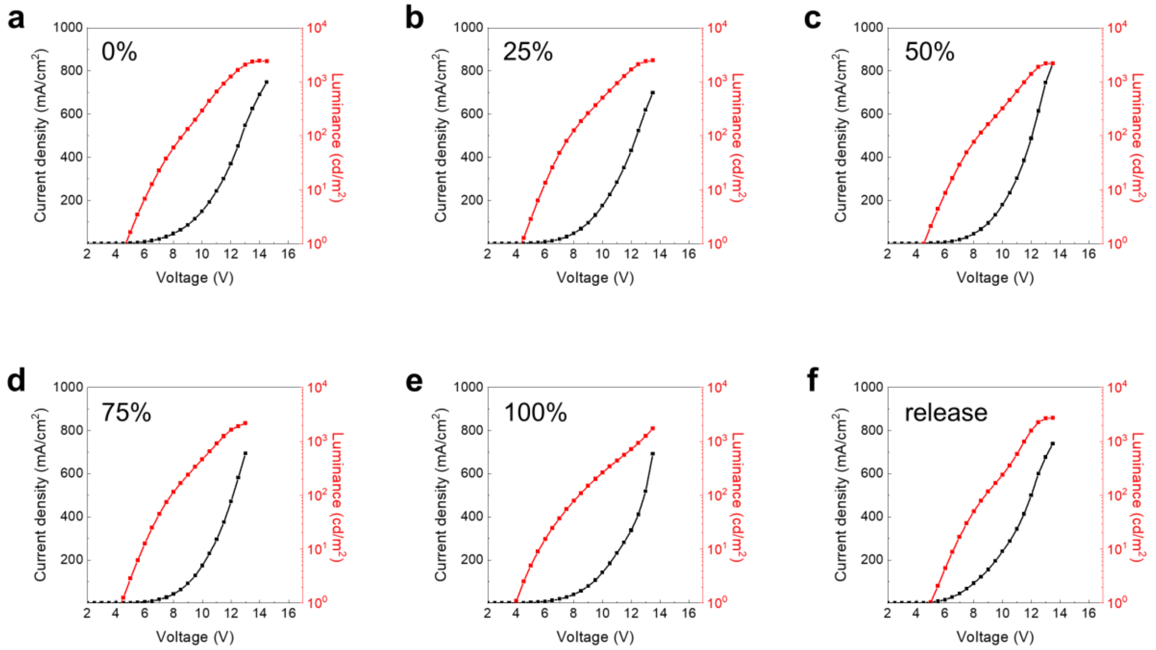

Fig. S32. Electroluminescence characteristics of PLED devices with stretched SP-red:SEBS blend films. The device structure is ITO/PEDOT:PSS/TFB/strained SP-red:SEBS/ZnO/Al. Current density-voltage-luminance curves are obtained with (a) 0%, (b) 25%, (c) 50%, (d) 75%, (e) 100% strained and (f) released SP-red:SEBS blend films in PLED devices.

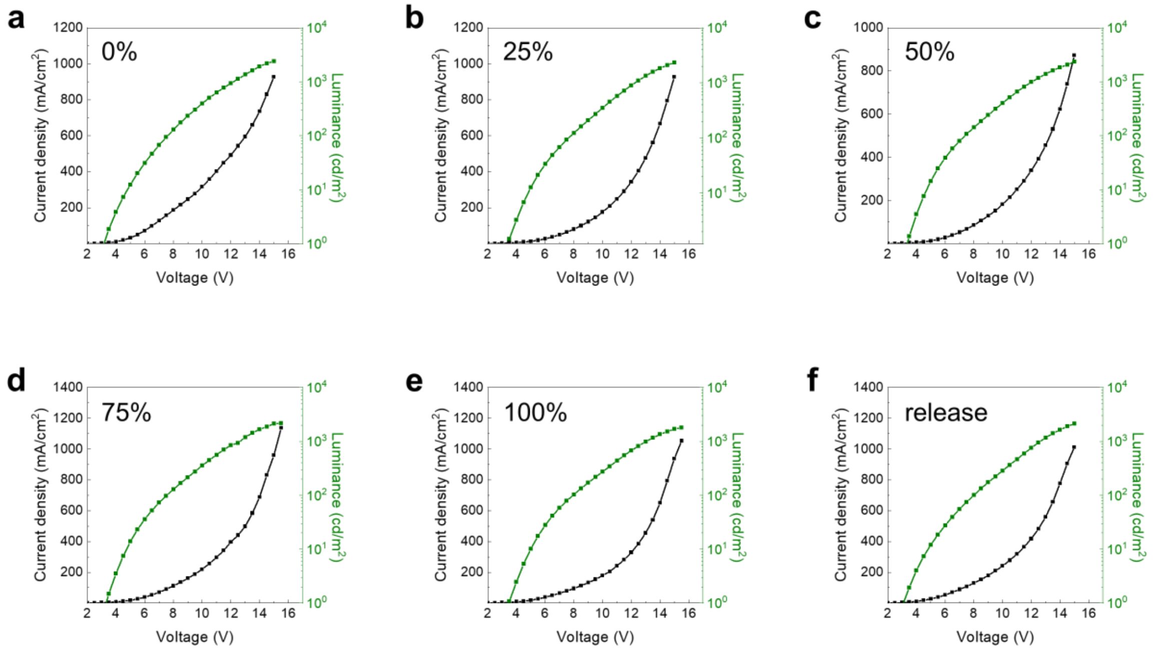

Fig. S33. Electroluminescence characteristics of PLED devices with stretched SP-green:SEBS blend films. The device structure is ITO/PEDOT:PSS/TFB/strained SP-green:SEBS/ZnO/Al. Current density-voltage-luminance curves are obtained with (a) 0%, (b) 25%, (c) 50%, (d) 75%, (e) 100% strained and (f) released SP-green:SEBS blend films in PLED devices.

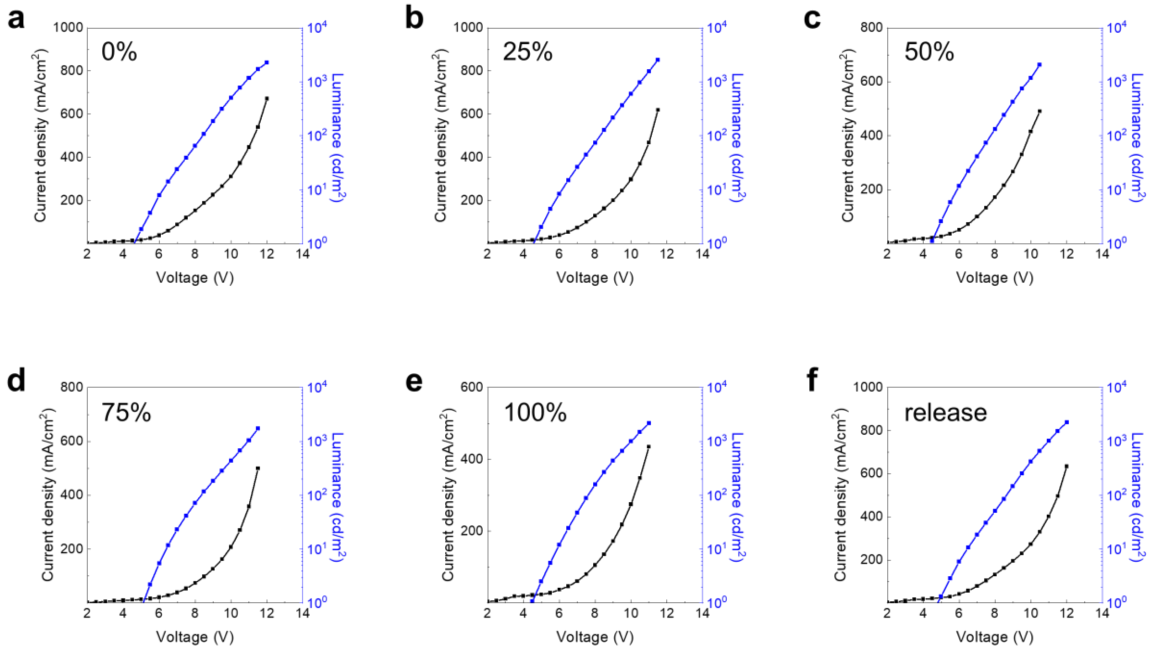

Fig. S34. Electroluminescence characteristics of PLED devices with stretched PFO:SEBS blend films. The device structure is ITO/PEDOT:PSS/TFB/strained PFO:SEBS/ZnO/Al. Current density-voltage-luminance curves are obtained with (a) 0%, (b) 25%, (c) 50%, (d) 75%, (e) 100% strained and (f) released PFO:SEBS blend films in PLED devices.

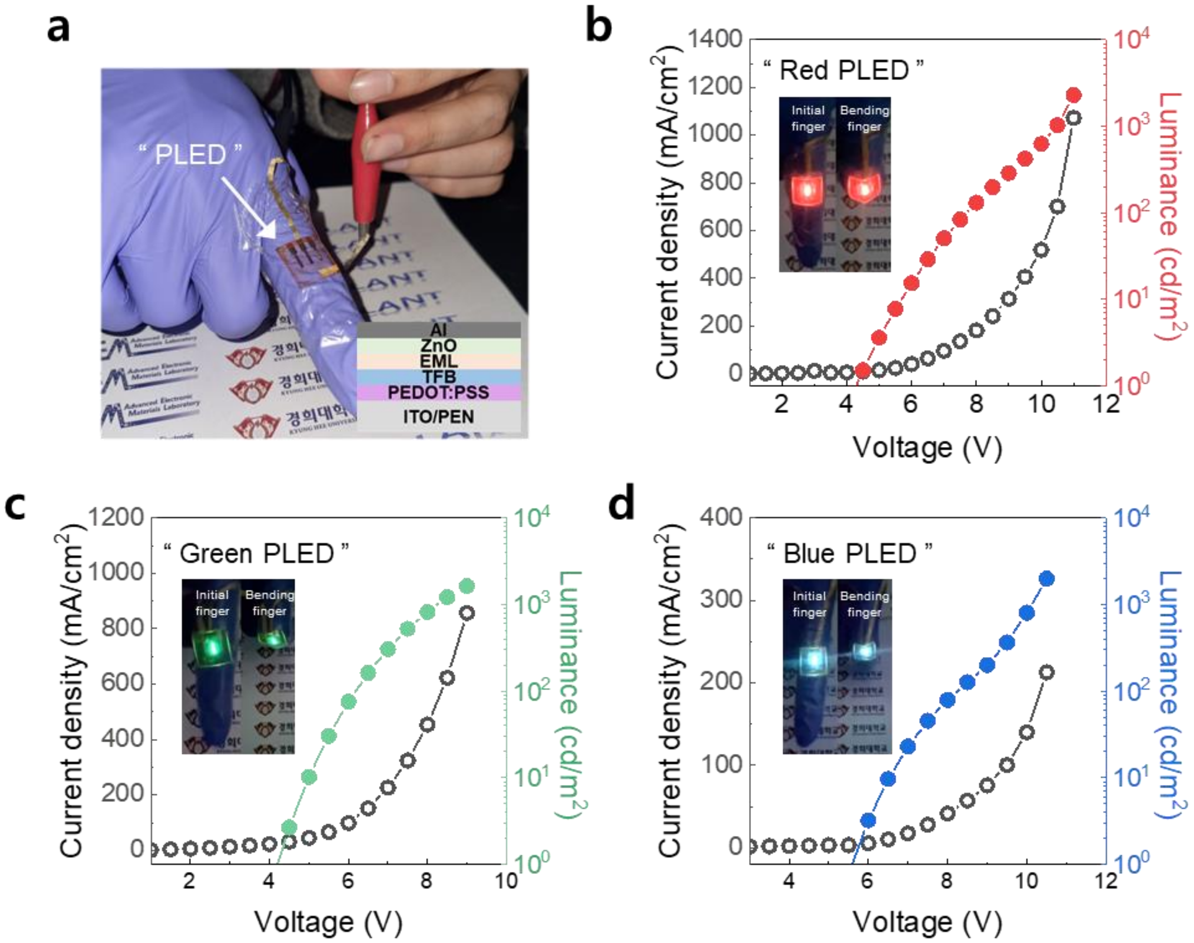

Fig. S35. Fabrication of flexible PLED devices. The flexible PLED devices were fabricated on (a) PEN substrate with PEN/ITO/PEDOT:PSS/TFB/stretchable EML films/ZnO/Al device structure. The (b) red, (c) green and (d) blue PLED devices can be operated stably (bending radius: 5 mm, bending angle: 100°).

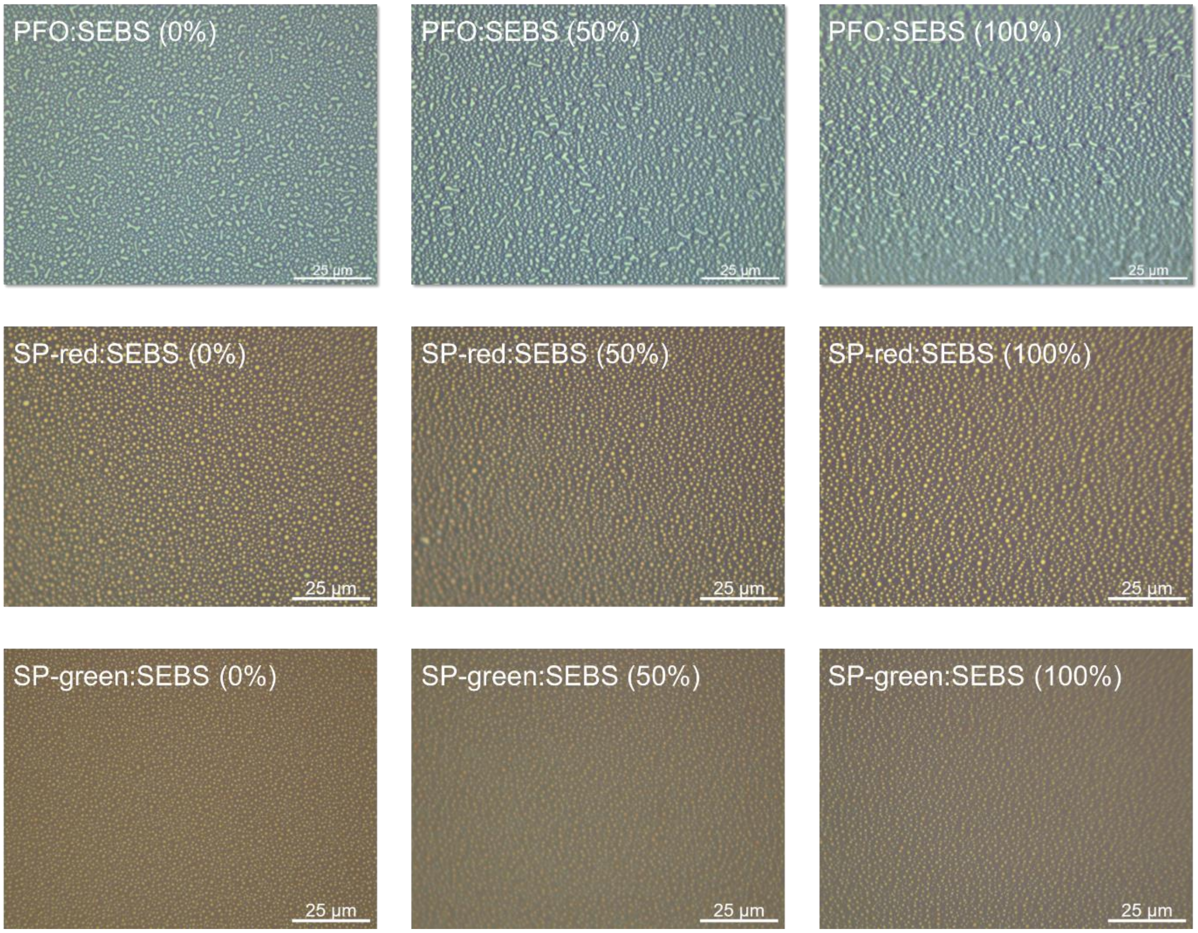

Fig. S36. OM images. PFO:SEBS (4:6), SP-red:SEBS (3:7) and SP-green:SEBS (2:8) blend films under different strain conditions.

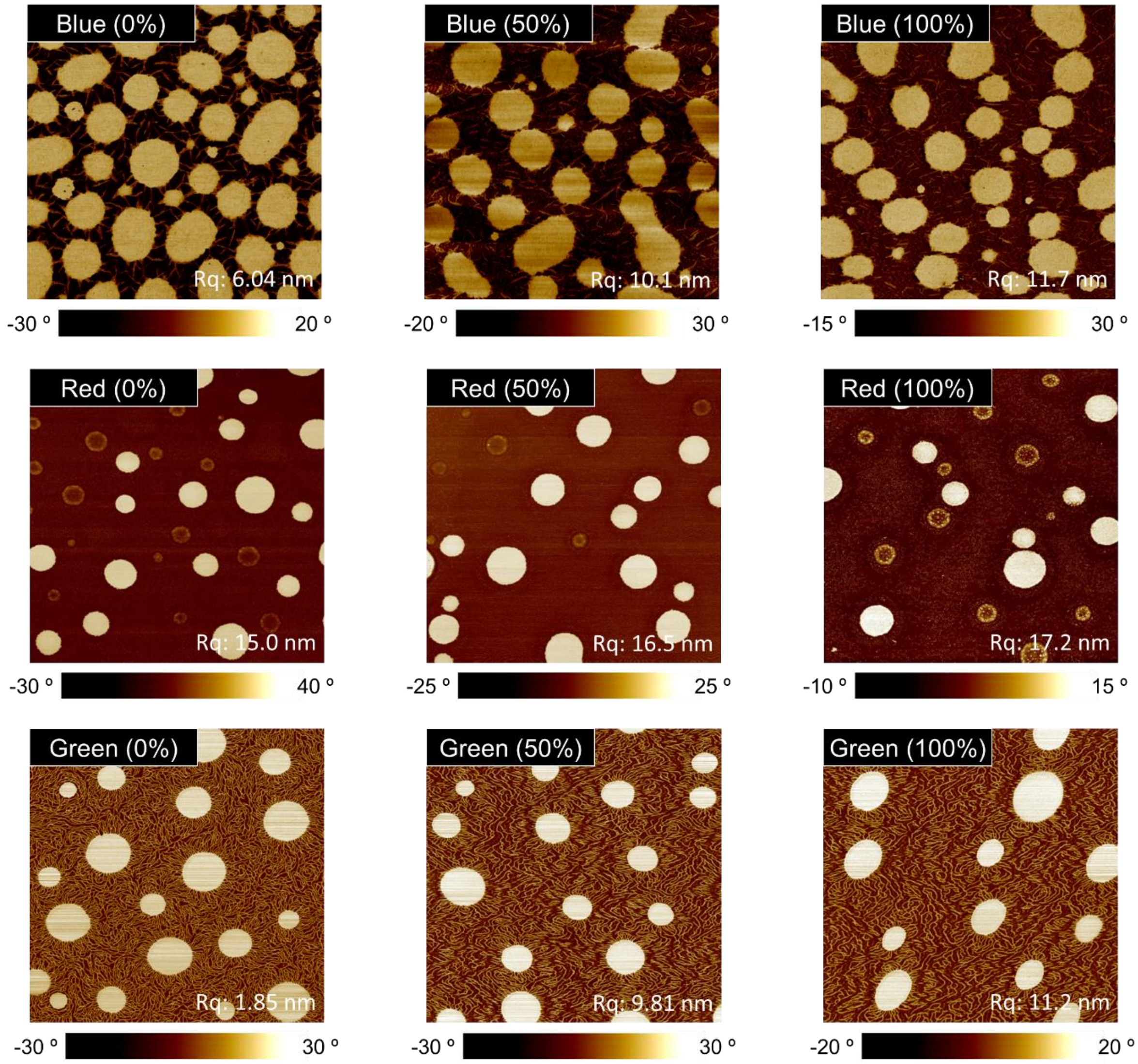

Fig. S37. Nanomorhology analysis on strain. AFM phase images of PFO:SEBS, SP-red:SEBS and SP-green:SEBS blend films under different strain conditions.

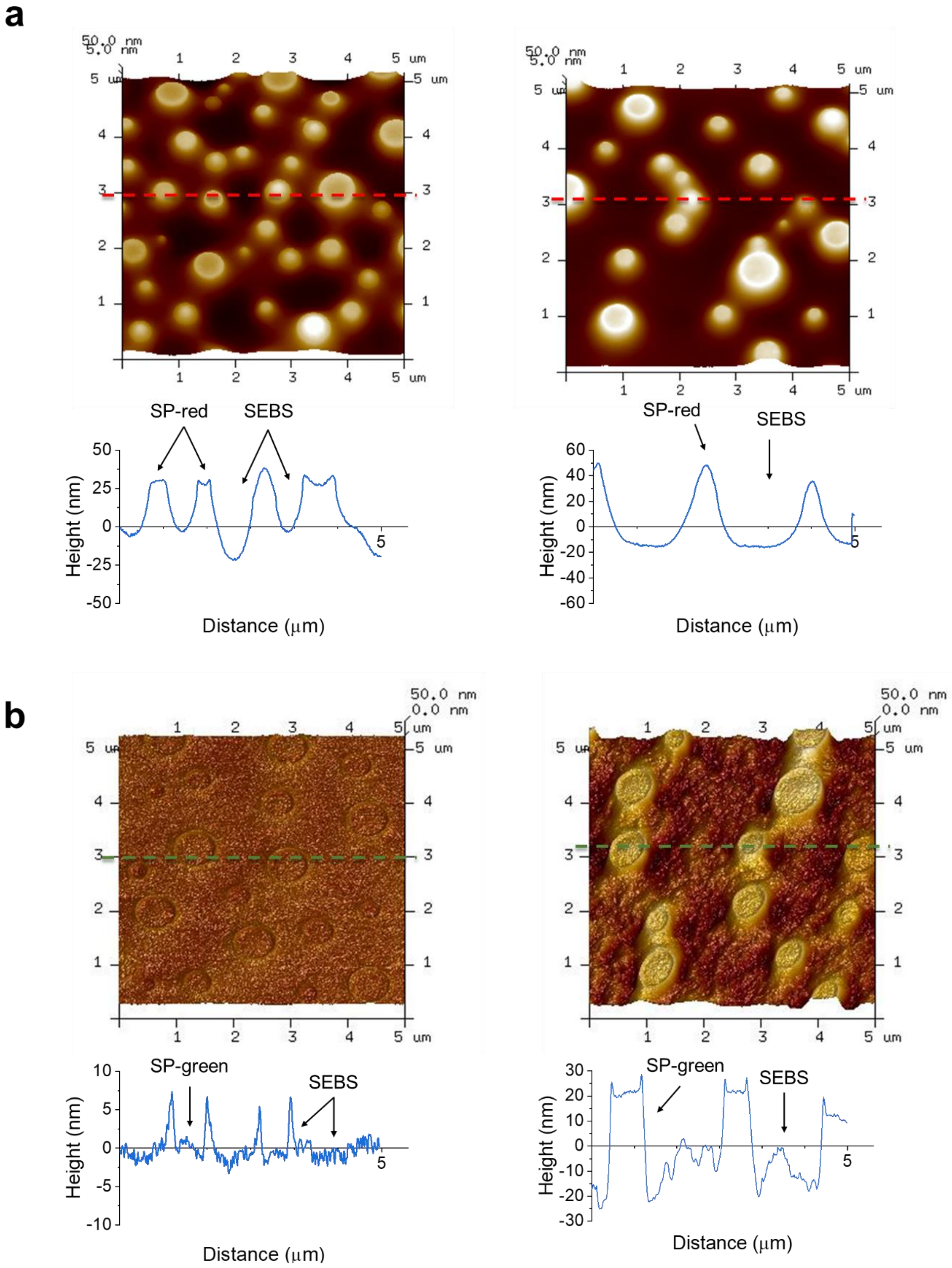

Fig. S38. 3D nanomorphology analysis. 3D AFM height images (up) with (a) SP-red:SEBS and (b) SP-green:SEBS blend films. The red and green lines on height images show cross-section point expressed in one dimensional height curves (down).

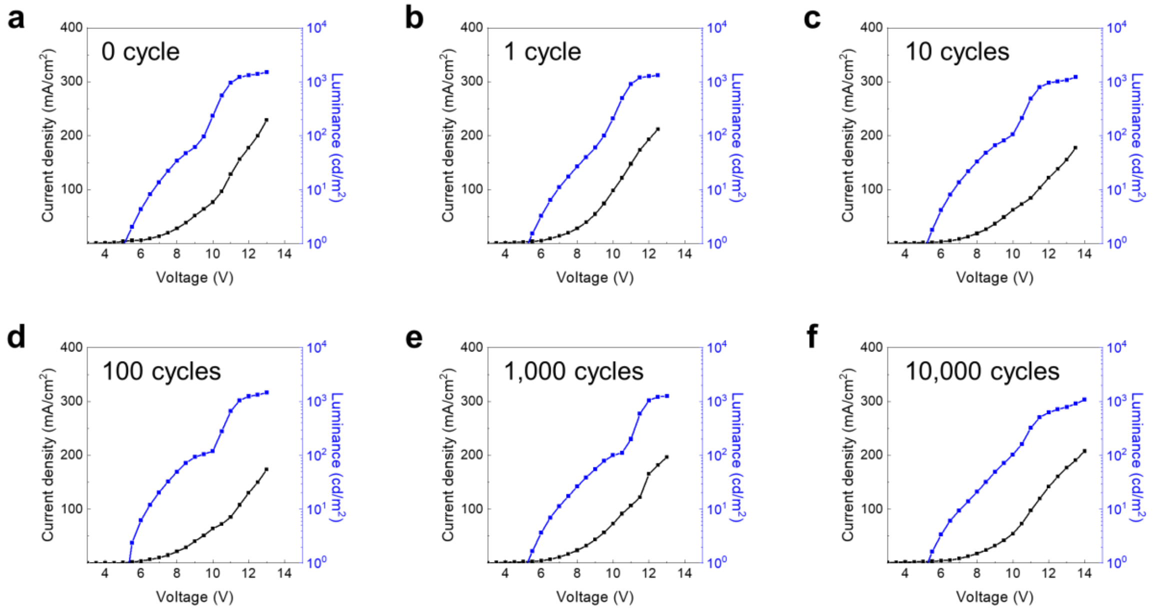

Fig. S39. Electroluminescence characteristics of PLED devices with PFO:SEBS films after stretching cycles at 25% strain. The device structure is ITO/PEDOT:PSS/TFB/released PFO:SEBS/ZnO/Al. Current density-voltage-luminance curves are obtained with (a) 0, (b) 1, (c) 10, (d) 100, (e) 1,000 and (f) 10,000 stretching cycles of PFO:SEBS films in PLED devices.

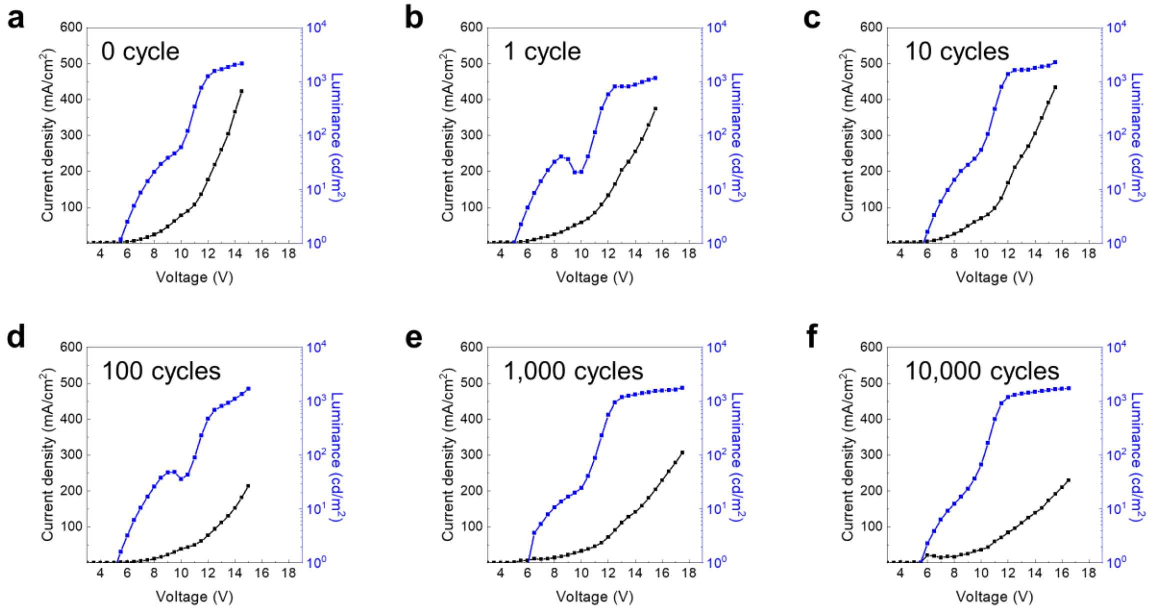

Fig. S40. Electroluminescence characteristics of PLED devices with PFO:SEBS films after stretching cycles at 50% strain. The device structure is ITO/PEDOT:PSS/TFB/released PFO:SEBS/ZnO/Al. Current density-voltage-luminance curves are obtained with (a) 0, (b) 1, (c) 10, (d) 100, (e) 1,000 and (f) 10,000 stretching cycles of PFO:SEBS films in PLED devices.

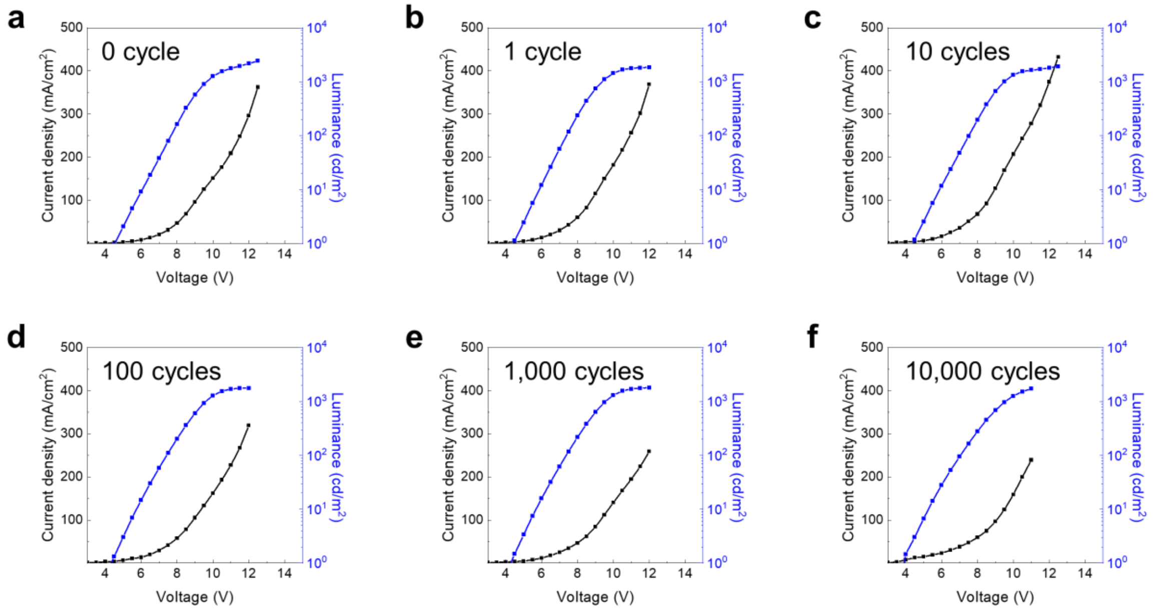

Fig. S41. Electroluminescence characteristics of PLED devices with PFO:SEBS film after stretching cycles at 75% strain. The device structure is ITO/PEDOT:PSS/TFB/released PFO:SEBS/ZnO/Al. Current density-voltage-luminance curves are obtained with (a) 0, (b) 1, (c) 10, (d) 100, (e) 1,000 and (f) 10,000 stretching cycles of PFO:SEBS films in PLED devices.

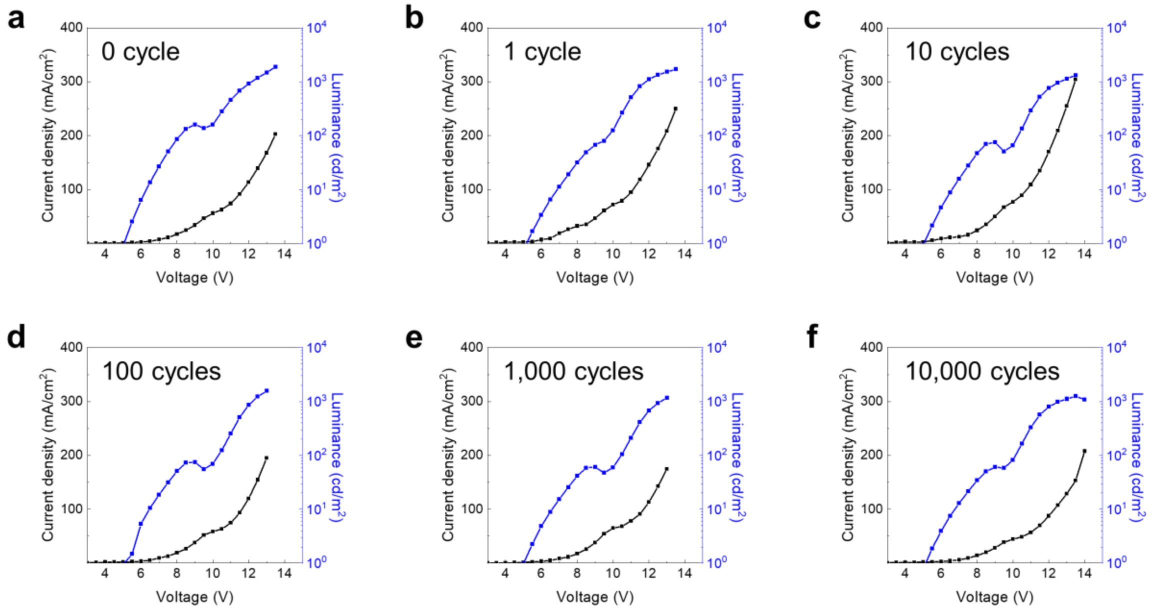

Fig. S42. Electroluminescence characteristics of PLED devices with PFO:SEBS films after stretching cycles at 100% strain. The device structure is ITO/PEDOT:PSS/TFB/released PFO:SEBS/ZnO/Al. Current density-voltage-luminance curves are obtained with (a) 0, (b) 1, (c) 10, (d) 100, (e) 1,000 and (f) 10,000 stretching cycles of PFO:SEBS films in PLED devices.

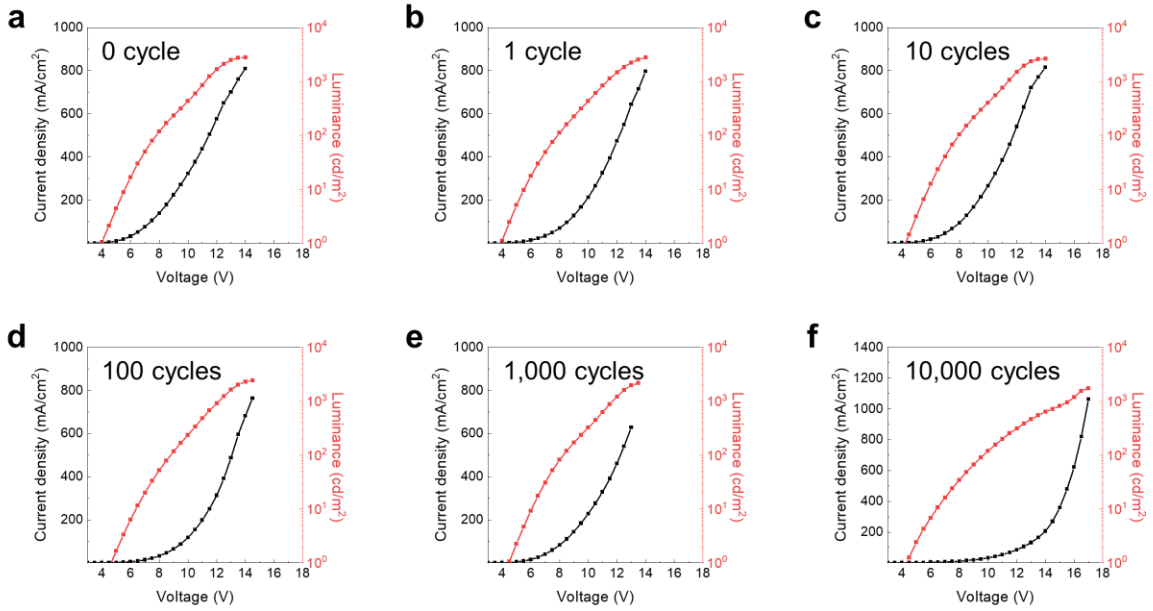

Fig. S43. Electroluminescence characteristics of PLED devices with SP-red:SEBS films after stretching cycles at 25% strain. The device structure is ITO/PEDOT:PSS/TFB/released SP-red:SEBS/ZnO/Al. Current density-voltage-luminance curves are obtained with (a) 0, (b) 1, (c) 10, (d) 100, (e) 1,000 and (f) 10,000 stretching cycles of SP-red:SEBS films in PLED devices.

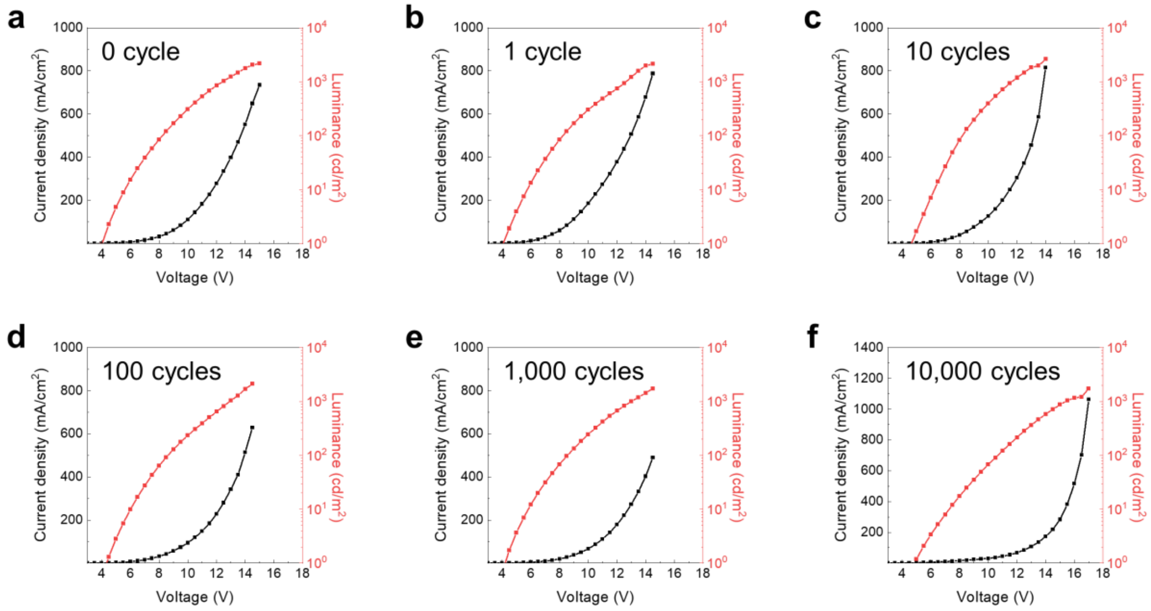

Fig. S44. Electroluminescence characteristics of PLED devices with SP-red:SEBS films after stretching cycles at 50% strain condition. The device structure is ITO/PEDOT:PSS/TFB/released SP-red:SEBS/ZnO/Al. Current density-voltage-luminance curves are obtained with (a) 0, (b) 1, (c) 10, (d) 100, (e) 1,000 and (f) 10,000 stretching cycles of SP-red:SEBS films in PLED devices.

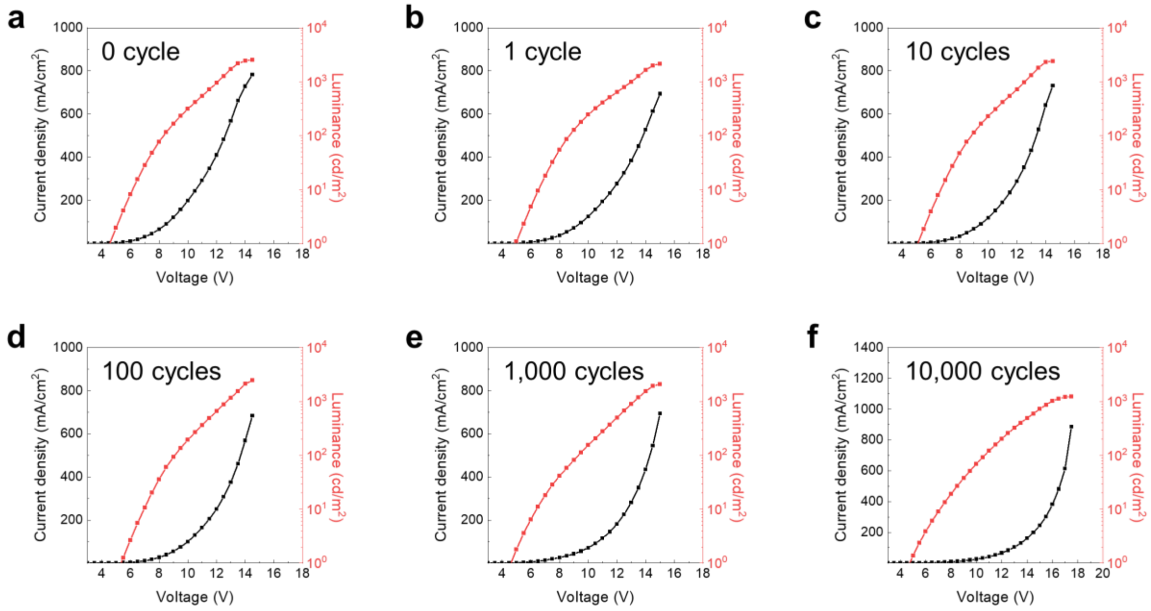

Fig. S45. Electroluminescence characteristics of PLED devices with SP-red:SEBS films after stretching cycles at 75% strain condition. The device structure is ITO/PEDOT:PSS/TFB/released SP-red:SEBS/ZnO/Al. Current density-voltage-luminance curves are obtained with (a) 0, (b) 1, (c) 10, (d) 100, (e) 1,000 and (f) 10,000 stretching cycles of SP-red:SEBS films in PLED devices.

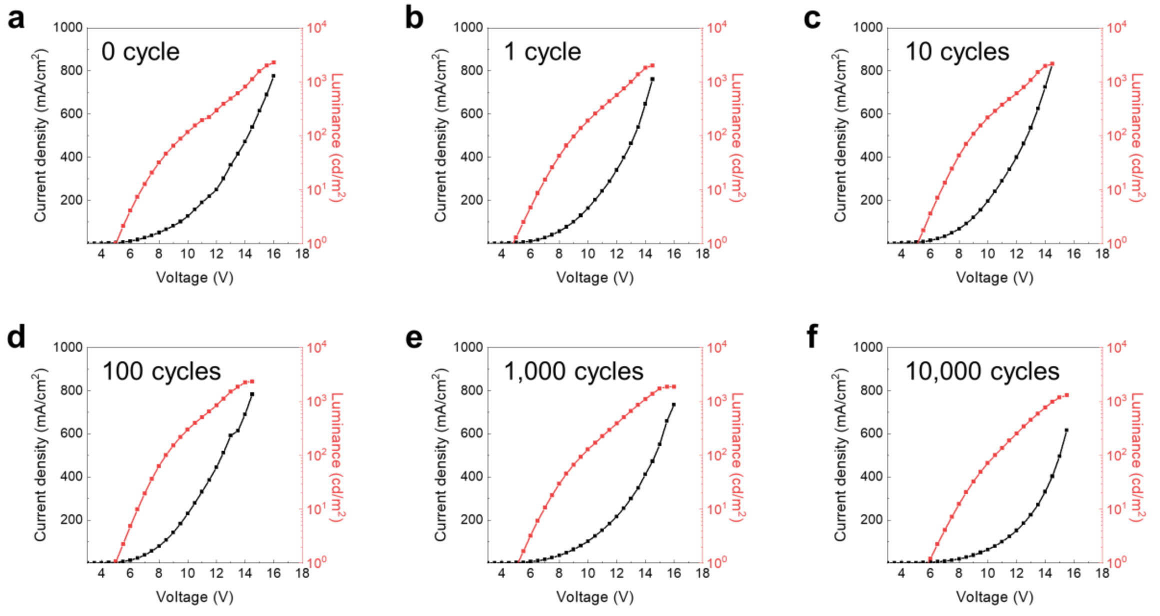

Fig. S46. Electroluminescence characteristics of PLED devices with SP-red:SEBS films after stretching cycles at 100% strain. The device structure is ITO/PEDOT:PSS/TFB/released SP-red:SEBS/ZnO/Al. Current density-voltage-luminance curves are obtained with (a) 0, (b) 1, (c) 10, (d) 100, (e) 1,000 and (f) 10,000 stretching cycles of SP-red:SEBS films in PLED devices.

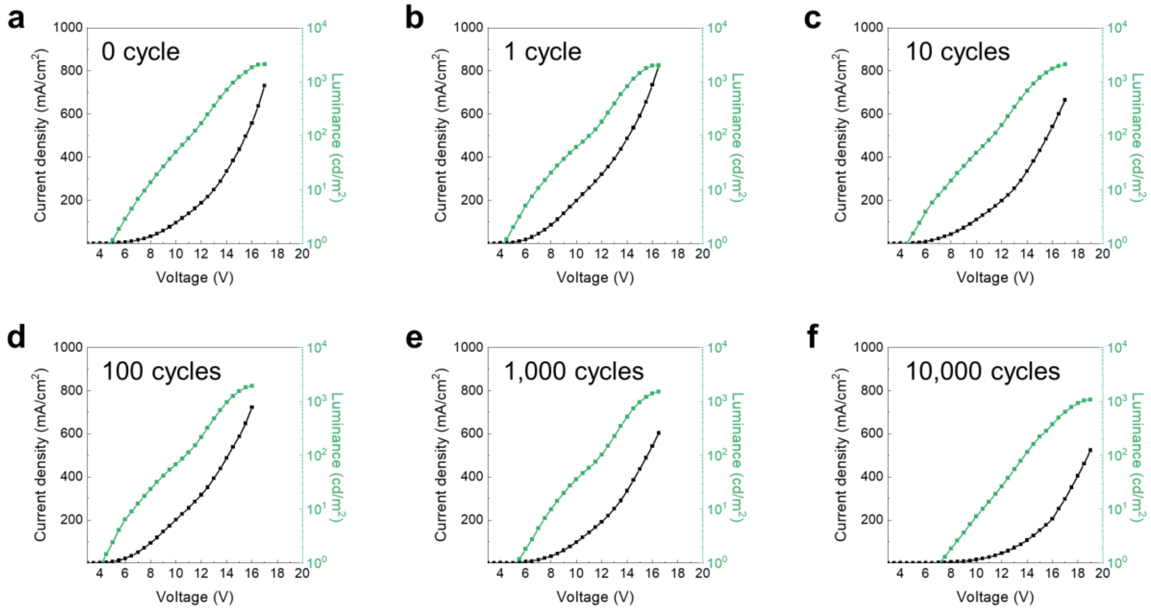

Fig. S47. Electroluminescence characteristics of PLED devices with SP-green:SEBS films after stretching cycles at 25% strain. The device structure is ITO/PEDOT:PSS/TFB/released SP-green:SEBS/ZnO/Al. Current density-voltage-luminance curves are obtained with (a) 0, (b) 1, (c) 10, (d) 100, (e) 1,000 and (f) 10,000 stretching cycles of SP-green:SEBS films in PLED devices.

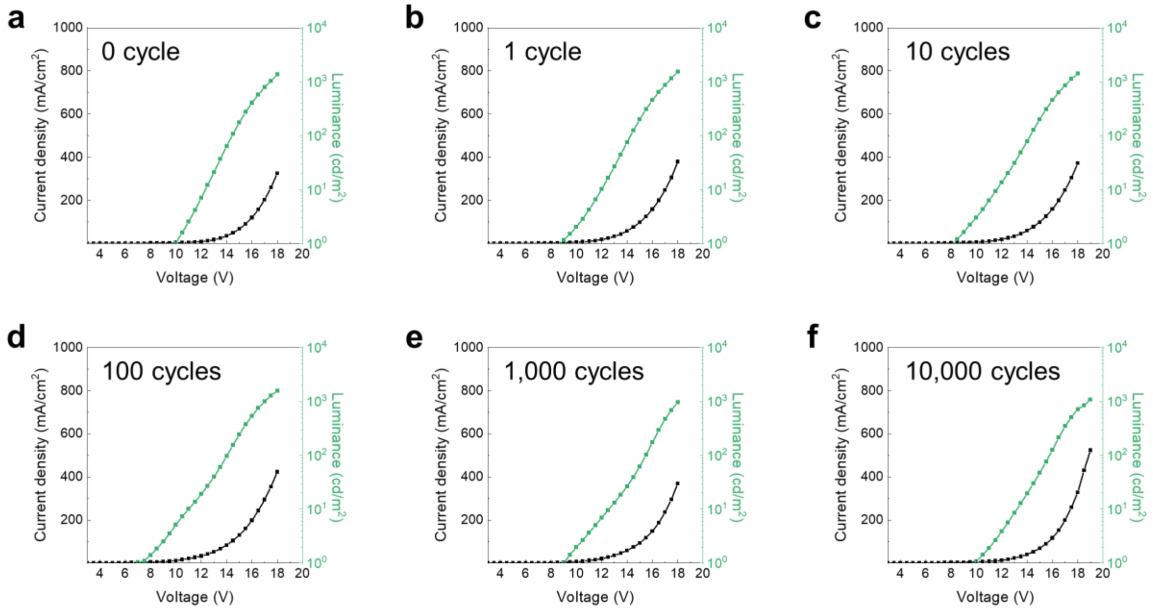

Fig. S48. Electroluminescence characteristics of PLED devices with SP-green:SEBS films after stretching cycles at 50% strain. The device structure is ITO/PEDOT:PSS/TFB/released SP-green:SEBS/ZnO/Al. Current density-voltage-luminance curves are obtained with (a) 0, (b) 1, (c) 10, (d) 100, (e) 1,000 and (f) 10,000 stretching cycles of SP-green:SEBS films in PLED devices.

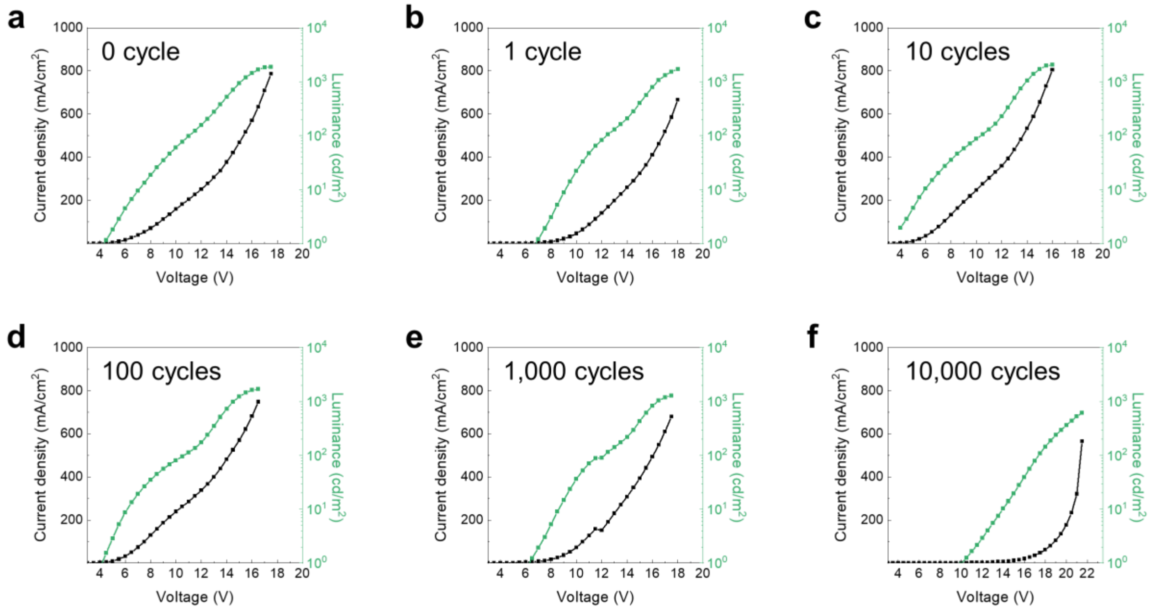

Fig. S49. Electroluminescence characteristics of PLED devices with SP-green:SEBS films after stretching cycles at 75% strain. The device structure is ITO/PEDOT:PSS/TFB/released SP-green:SEBS/ZnO/Al. Current density-voltage-luminance curves are obtained with (a) 0, (b) 1, (c) 10, (d) 100, (e) 1,000 and (f) 10,000 stretching cycles of SP-green:SEBS films in PLED devices.

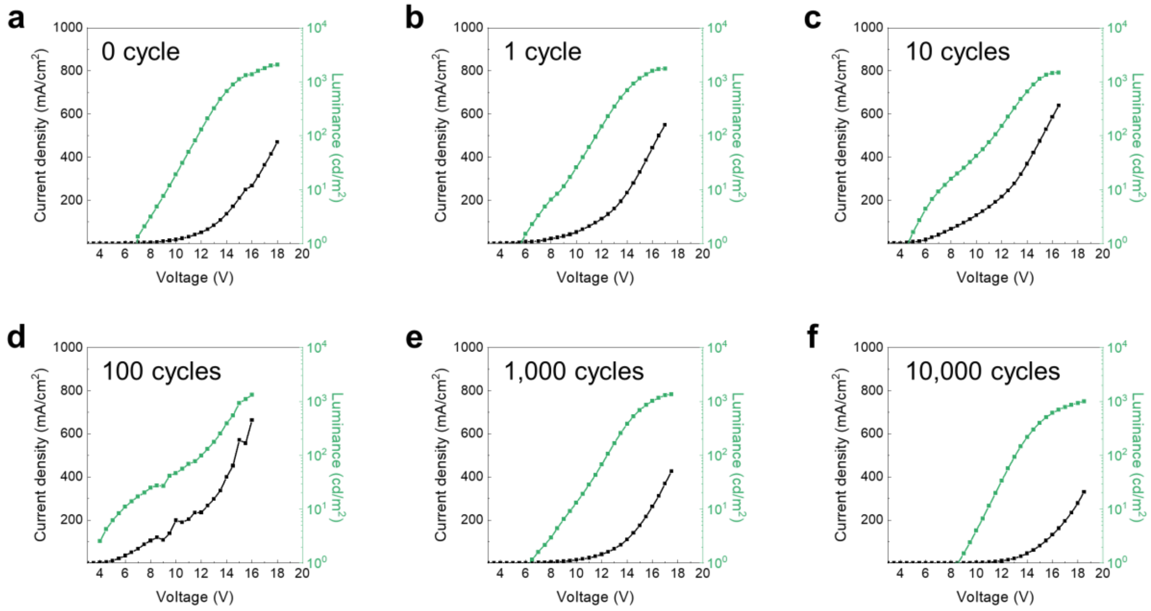

Fig. S50. Electroluminescence characteristics of PLED devices with SP-green:SEBS films after stretching cycles at 100 % strain. The device structure is ITO/PEDOT:PSS/TFB/released SP-green:SEBS/ZnO/Al. Current density-voltage-luminance curves are obtained with (a) 0, (b) 1, (c) 10, (d) 100, (e) 1,000 and (f) 10,000 stretching cycles of SP-green:SEBS films in PLED devices.

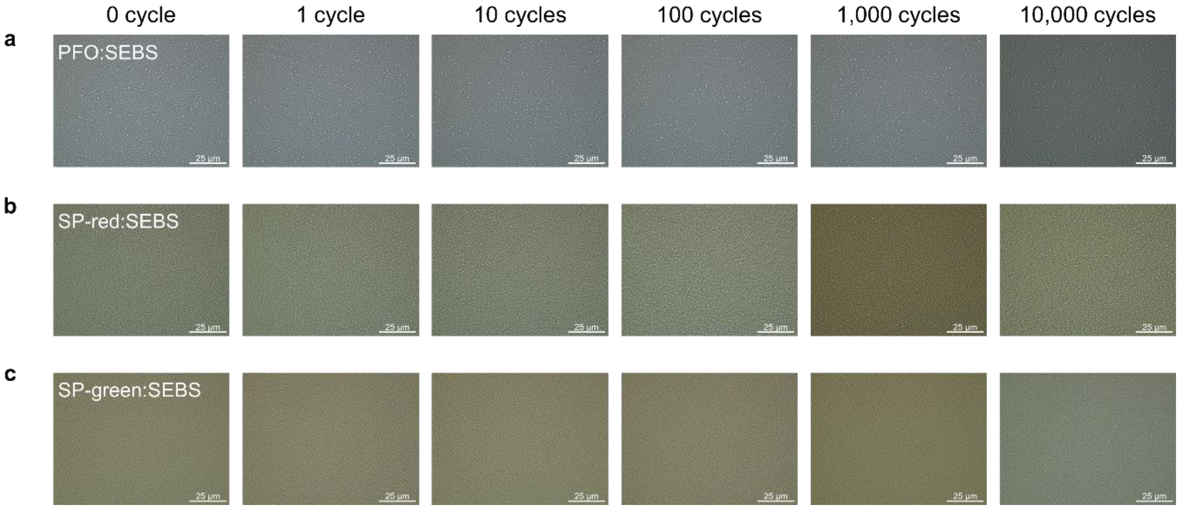

Fig. S51. Stretching durability test. OM images after multiple stretching cycles (from 0 to 10,000 times) with (a) PFO:SEBS, (b) SP-red:SEBS and (c) SP-green:SEBS blend films. All cycle tests were performed at 100% strain.

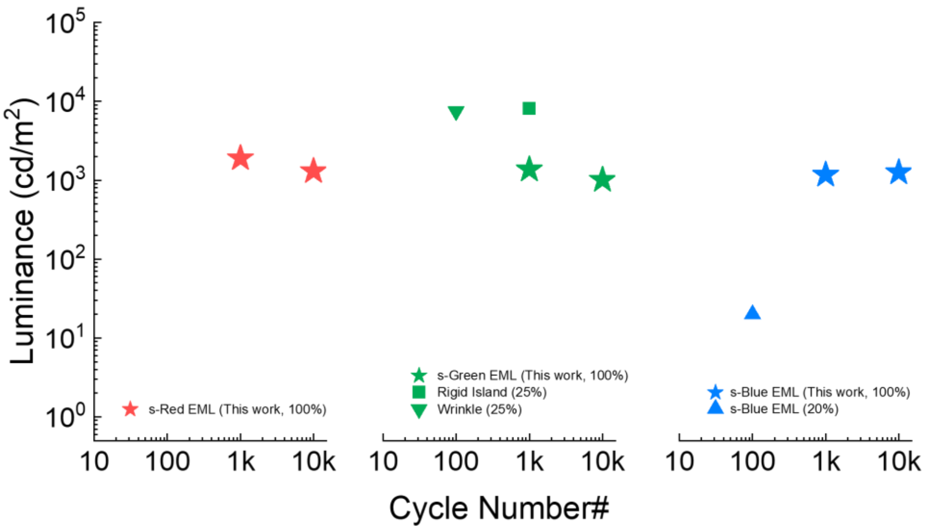

Fig. S52. Comparison of luminance of this work. Comparison of luminance of stretchable red, green, and blue light-emitting films and previously reported works according to number of stretching cycles at different strains.

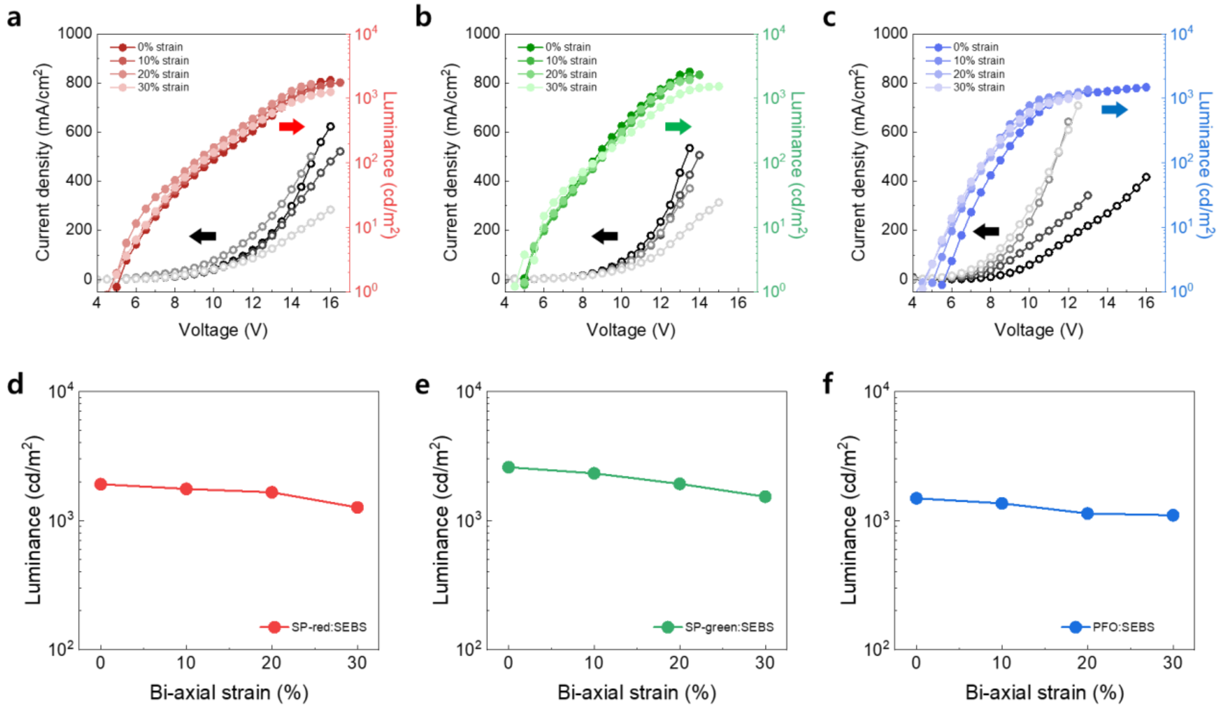

Fig. S53. Bi-axial stretching test of the EML film. J-V-L curves and luminance values of (a,d) SP-red:SEBS, (b,e) SP-green:SEBS and (c,f) PFO:SEBS blend films on various bi-axial strain.

Table S1. The surface energies (γ) and interaction parameters (χ) of light emitting films.

		Contact Angle (º)		Surface Energy (mJ/m^2)			Interaction parameter (χ)
		DI-water	Diiodomethane	γ_{total}	γ_{polar}	$\gamma_{dispersion}$	
**Neat**	PFO	103.2	46.4	36.3	36.3	0.82×10^{-2}	-
	SP-red	91.6	34.8	41.8	41.4	0.45	-
	SP-green	96.7	41.7	38.7	38.3	0.40	-
**Blend**	PFO:SEBS	95.6	50.2	34.1	33.4	0.77	0.05K
	SP-red:SEBS	103.5	49.9	34.3	34.3	0.59×10^{-3}	0.17K
	SP-green:SEBS	103.1	48.9	34.9	34.8	0.39×10^{-2}	0.45K

Table S2. The solubility parameters for calculating relative energy density (RED).

Material	δ_D	δ_P	δ_H	δ_r	sphere radius (R_0)
PFO	18.55	2.8	4.51	19.81	4.1
SEBS	16.388	0.198	0	16.39	8

δ_D: dispersive solubility parameter, δ_P: polar solubility parameter, δ_H: hydrogen bonding paramter, δ_r: Hansen solubility parameter

Table S3. Comparison of operating performance and color purity with CIE 1931 coordinates.

Light Color	Turn-on voltage (V_{on})	CIE 1931 Coordinates at V_{on}	Maximum luminance (L_{max})	Operating voltage at L_{max}	CIE 1931 Coordinates at L_{max}	[Ref.]
Red	2.82 V	(0.60, 0.37)	4,061 cd/m^2	10.5 V	(0.64, 0.36)	This work
Green	2.65 V	(0.28, 0.54)	2,418 cd/m^2	10.5 V	(0.28, 0.56)	This work
Blue	3.71 V	(0.25, 0.28)	5,823 cd/m^2	9.5 V	(0.29, 0.38)	This work
Blue	~ 6.4 V	n/a	117 cd/m^2	11.5 V	(0.15, 0.06)	[24]
Blue	2 V	n/a	~ 70 cd/m^2	10 V	n/a	[25]
Blue	~ 10 V	n/a	331 cd/m^2	19 V	n/a	[26]
Blue	9.3 V	n/a	3,274 cd/m^2	28.5 V	(0.15, 0.12)	[27]
Red	~ 3 V	n/a	~ 7,100 cd/m^2	9.5 V	n/a	[28]
Green	~ 4.5 V	n/a	~ 3,200 cd/m^2	12 V	n/a	[28]
Blue	~ 4.5 V	n/a	~ 1,900 cd/m^2	7.5 V	n/a	[28]

V_{on}: Turn-on voltage at 1 cd/m^2 luminance, L_{max}: Maximum luminance value, (~): approximate value, n/a: not applicable

Movie S1. In-situ measurement of the three primary color RGB PLEDs for bending and on-human finger operations.
